# The effect of plant active substances on cognitive function in healthy older adults: a systematic review and network meta-analysis of randomized controlled trials

**DOI:** 10.3389/fphar.2025.1672171

**Published:** 2026-01-20

**Authors:** Xueyan Feng, Shuangbo Fan, Fengqin Wei

**Affiliations:** 1 College of Traditional Chinese Medicine, Shandong University of Traditional Chinese Medicine, Jinan, China; 2 Department of Pain Management, Shandong Second Provincial General Hospital, Jinan, China

**Keywords:** age related cognitive decline, cognitive function, healthy olderadults, network meta-analysis, plant active substances

## Abstract

**Background:**

With the accelerating global population aging, age-related cognitive decline has become a significant health concern for the older adults. The limited efficacy and common side effects of pharmacological interventions have made the exploration of safe non-pharmacological strategies an urgent need. Plant active substances have drawn much attention due to their multi-target neuroprotective properties, there is a lack of systematic comparative research on cognitive function in healthy older adults.

**Objective:**

To assess the effects of plant active substances on various domains of cognitive function in healthy older adults through a network meta-analysis (NMA).

**Methods:**

Comprehensive searches were conducted in Embase, PubMed, Cochrane Library, and Web of Science (up to 31 December 2024). Primary outcomes included learning and memory, complex attention, executive function, language, and perceptual-motor skills. NMA was performed using Stata 15.1, with cumulative ranking curve area (SUCRA) for intervention ranking; consistency and publication bias were examined.

**Results:**

After screening, 25 eligible studies with 1861 healthy older adults, evaluating 10 plant active substances, were included. Specifically, 23 studies included learning and memory functions, 18 studies included complex attention, 16 studies included executive functions, 4 studies included language functions, and 3 studies included perceptual-motor functions. The research results showed that based on the SUCRA values, raisins (95.1%) and tart cherry (89.5%) were the most likely to be the best intervention for improving learning and memory functions. The bacopa monnieri compound (91.3%), curcumin (89.3%), and tart cherry (88.9%) ranked in the top three in the executive function domain. Bacopa monnieri compound (93%) and raisin (80.7%) ranked in the language function category. Guarana (90.3%) had the highest probability in the perceptual-motor domain. The intervention effects on complex attention functions were generally limited.

**Conclusion:**

The NMA results indicate that in terms of learning and memory functions, raisin and tart cherry ranked higher; in terms of executive functions, the bacopa monnieri compound demonstrated a relatively better intervention effect, providing an important basis for non-drug interventions for cognitive health in the healthy older adults. Future research should focus on long-term safety, dosage optimization, and synergistic mechanisms to promote functional food development.

**Systematic Review Registration:**

https://www.crd.york.ac.uk/PROSPERO/, identifier CDR420251032046.

## 1 Introduction

The growing global aging population has made the maintenance of cognitive function in older adults a critical public health issue. According to data from the World Health Organization, by 2030, one in six people worldwide will be aged 60 and above (Rudnicka et al., 2020). In this context, Age-Related Cognitive Decline (ARCD) is becoming a central issue threatening the ability of older adults to live independently (Olejnik et al., 2025), and the global prevalence of dementia is expected to double over the next 25 years (Gausemel and Filkuková, 2025). It should be emphasized that ARCD denotes a physiological decline in cognitive function associated with advancing age; its severity does not meet the diagnostic threshold for mild cognitive impairment (MCI), which represents a transitional state between normal aging and dementia (Roberts and Knopman, 2013). However, current pharmacological treatments, such as cholinesterase inhibitors, have limited efficacy and are associated with side effects (Sharma, 2019). Therefore, exploring safe non-pharmacological intervention strategies is of significant clinical and societal importance.

In recent years, natural plant active substances, such as polyphenols, flavonoids, and terpenoids, have garnered widespread attention due to their multi-target mechanisms of action and potential neuroprotective properties (Baranowska-Wojcik et al., 2025; Lorca et al., 2023). The existing meta-analysis studies mostly focus on the impact of individual plant active substances on cognitive function. The conclusions of these studies are often inconsistent due to the heterogeneity of the research population, intervention dosage, treatment duration, and cognitive assessment tools. They also cannot be directly compared among different components, making it difficult to determine which component is more advantageous. Network Meta Analysis (NMA) can integrate direct and indirect evidence under a unified framework, enabling comprehensive comparison and probability ranking of multiple intervention measures, and providing methodological support for selecting the optimal intervention. Furthermore, most studies focus on cognitive impairment interventions in pathological conditions (Hack et al., 2023; Pagotto et al., 2024; Travica et al., 2020), leaving a gap in preventive research for healthy older adults populations. Accordingly, this study employed a network meta-analysis to systematically evaluate the cognitive effects of the following plant active substances in healthy older adults: Bacopa monnieri (L.) Wettst. (Plantaginaceae; Bacopae monnieri herba), Paullinia cupana Kunth (Sapindaceae; Paulliniae cupanae semen), Ginkgo biloba L. (Ginkgoaceae; Ginkgo folium), Salvia officinalis L. (Lamiaceae; Salviae officinalis folium), Coffea arabica L. (Rubiaceae; Coffeae semen), Avena sativa L. (Poaceae; Avenae herba), Curcuma longa L. (Zingiberaceae; Curcumae longae rhizoma), Vaccinium corymbosum L. (Ericaceae; Vaccinii corymbosi fructus), Vitis vinifera L. (Vitaceae; Uvae passae fructus), and Prunus cerasus L. (Rosaceae; Cerasi acidi fructus) (see Table 1). The findings are expected to provide a theoretical basis for non-pharmacological interventions in older adults cognitive health and lay the scientific foundation for the development of plant-based functional foods.

**TABLE 1:** Botanical identity of plant active substances.

Common name	Latin name	Family	Plant part used
Bacopa monnieri	Bacopa monnieri (L.) wettst	Plantaginaceae	Bacopae monnieri herba
Guarana	Paullinia cupana kunth	Sapindaceae	Paulliniae cupanae semen
Ginkgo biloba	Ginkgo biloba L	Ginkgoaceae	Ginkgo folium
Salvia	Salvia officinalis L	Lamiaceae	Salviae officinalis folium
Caffeine	Coffea arabica L	Rubiaceae	Coffeae semen
Wild green oat	Avena sativa L	Poaceae	Avenae herba
Curcumin	Curcuma longa L	Zingiberaceae	Curcumae longae rhizoma
Blueberry	Vaccinium corymbosum L	Ericaceae	Vaccinii corymbosi fructus
Tart cherry	Prunus cerasus L	Rosaceae	Cerasi acidi fructus
Grape	Vitis vinifera L	Vitaceae	Vitis viniferae fructus
Raisin	Vitis vinifera L	Vitaceae	Uvae passae fructus

Common names are cited directly from the original RCT, publications.

## 2 Methods

The implementation of the meta-analysis and systematic review strictly adhered to the Preferred Reporting Items for Systematic Reviews and Meta-Analyses (PRISMA) guidelines and was registered with PROSPERO (registration number: CDR420251032046).

### 2.1 Search strategy

We systematically searched four databases: Embase, PubMed, Cochrane Library, and Web of Science, covering the period from their inception until 31 December 2024. The search strategy was based on the PICTOS framework, as follows: (P) Population: healthy older adults; (I) Intervention: plant active substances; (C) Comparison: placebo; (T) Timing: no restriction on intervention duration; (O) Outcome:cognitive function scores; (S) Study type: randomized controlled trials. A detailed example of the search strategy for PubMed can be found in Supplementary Table S1.

### 2.2 Inclusion and exclusion criteria

The inclusion criteria for this meta-analysis, as defined within the PICTOS framework, are as follows: (1) clinical randomized controlled trials; (2) study population: healthy older adults; This study defines “healthy older adults” as follows:①Age≥50 years; ②No history of dementia, Alzheimer’s disease, mild cognitive impairment or other neurological and mental disorders; ③Received a cognitive status assessment before enrollment and was confirmed to have no subjective cognitive decline. (3) intervention: treatment with different plant active substances; (4)outcomes: one or more of the following cognitive functions—attention, executive function, learning and memory, language, perceptual motor skills, and social cognition; (5) The trial reported the duration of the intervention.

Exclusion criteria: (1) study population does not include healthy older adults, such as patients, children, and young people. (2) non-clinical experimental studies; (3) conference proceedings, clinical guidelines, animal studies, cell studies, review articles, and meta-analyses; (4) incomplete or unpublished data; (5) studies without a control group.

### 2.3 Study selection

The literature screening process was conducted by two researchers at different stages. Initially, all database search results were exported to Endnote software to remove duplicate references. The screening process was then carried out in two stages. In the first stage, titles and abstracts were reviewed to exclude articles that did not meet the inclusion criteria. In the second stage, full-text articles of those passing the initial screening were reviewed to determine the final studies for inclusion. This process was carried out independently by two researchers, and any disagreements were resolved through discussion with a third researcher.

### 2.4 Data extraction

Data extraction was independently performed by two authors based on the final list of included studies. Any disagreements regarding the extraction of data were resolved through discussion with a third researcher. The recorded study characteristics included: (1) first author; (2) year of publication; (3) country; (4) sample size; (5) mean age of participants; (6) duration and dosage of the intervention; and (7) outcomes used to assess cognitive function levels. Multi-indicator processing rule: When several instruments were available for the same cognitive domain, the one most frequently employed was selected; if the test time points are different, the result with the longest time window should be taken to reflect the sustained effect.

### 2.5 Quality assessment

Two researchers independently assessed the risk of bias (ROB) in the included randomized controlled trials (RCTs) using the Cochrane Handbook version 6.5 tool. The following seven domains were considered: random sequence generation; allocation concealment via blinding of participants and personnel; incomplete outcome data; selective reporting; and other sources of bias. The trials were classified into three levels of ROB based on the number of domains with either high or unclear risk of bias. Low risk of bias: the trial was rated as low risk in all domains for the outcome. Some concerns: the trial was rated as raising some concerns in at least one domain, but not as high risk in any domain. High risk of bias: the trial was rated as high risk in at least one domain, or had multiple domains raising concerns to an extent that substantially undermines confidence in the result (Lunny et al., 2025).

### 2.6 Data analysis

This study focuses on continuous variables, represented by means and standard deviations (SD). For continuous variable analysis, we employed the mean difference (MD), which is the absolute difference between the means of the treatment and control groups (based on the same scale), or the standardized mean difference (SMD), which is the ratio of the difference between group means to the standard deviation of the outcome (suitable for integrating trial data from different scales). A 95% confidence interval (CI) was used for comprehensive analysis. Given the potential heterogeneity between studies, a random-effects model was chosen instead of a fixed-effects model to enhance the reliability of the analysis results (Jackson et al., 2011).

The study strictly adhered to the PRISMA NMA guidelines and utilized Stata (version 15.1) software to perform network meta-analysis (NMA) within a Bayesian framework, employing Markov chain Monte Carlo simulations for data aggregation (Moher et al., 2015; Shim et al., 2017; Vats et al., 2019). The consistency between indirect and direct comparisons was evaluated using the node-splitting method, with calculations performed via Stata commands. A p-value greater than 0.05 was considered as evidence of consistency (Salanti et al., 2011). The transitivity assumption is evaluated by comparing the similarities in population characteristics such as age, gender, and health status among the included studies, ensuring the comparability among the various studies. Interventions were ranked based on the surface under the cumulative ranking curve (SUCRA), where a higher SUCRA value indicates a better ranking. The SUCRA value represents the ranking probability, and its interpretation requires caution, as its clinical significance depends on the magnitude of actual differences between interventions (Marotta et al., 2020). To identify the risk of publication bias due to small-scale studies, a network funnel plot was constructed, with symmetry visually assessed to ensure the scientific rigor and objectivity of the results (Chaimani et al., 2013). Additionally, we used CINeMA (Confidence In Network Meta - Analysis) to draw the network plot. The line thickness mainly depends on the number of studies; The colors red, green, and yellow of the nodes and lines primarily represent the proportions of high, low, and unclear risk of bias, respectively (Nikolakopoulou et al., 2020; Papakonstantinou et al., 2020).

### 2.7 Assessment of reporting quality for phytochemical characterization

To enhance the transparency and reproducibility of reporting on the composition of plant active substances, extraction processes, and quality information in this study, we referred to the ConPhyMP consensus guidelines and its online tool (https://ga-online.org/best-practice/) to report the composition and processing of the preparations (Heinrich and Jalil, 2023; Heinrich et al., 2022). Two investigators (XF and SF) independently assessed the reporting quality of the included literature by verifying each item using the ConPhyMP checklist. Discrepancies were resolved through discussion or by consulting a third party (FW). In addition to completing the core ConPhyMP checklist (see Supplementary Table S2, S3), we separately summarized the species, medicinal parts used, extraction processes, and dosage ranges for each plant active substance (see Supplementary Table S4). This provides traceable data to facilitate future replication or safety assessments.

## 3 Results

### 3.1 Literature screening

**FIGURE 1:**
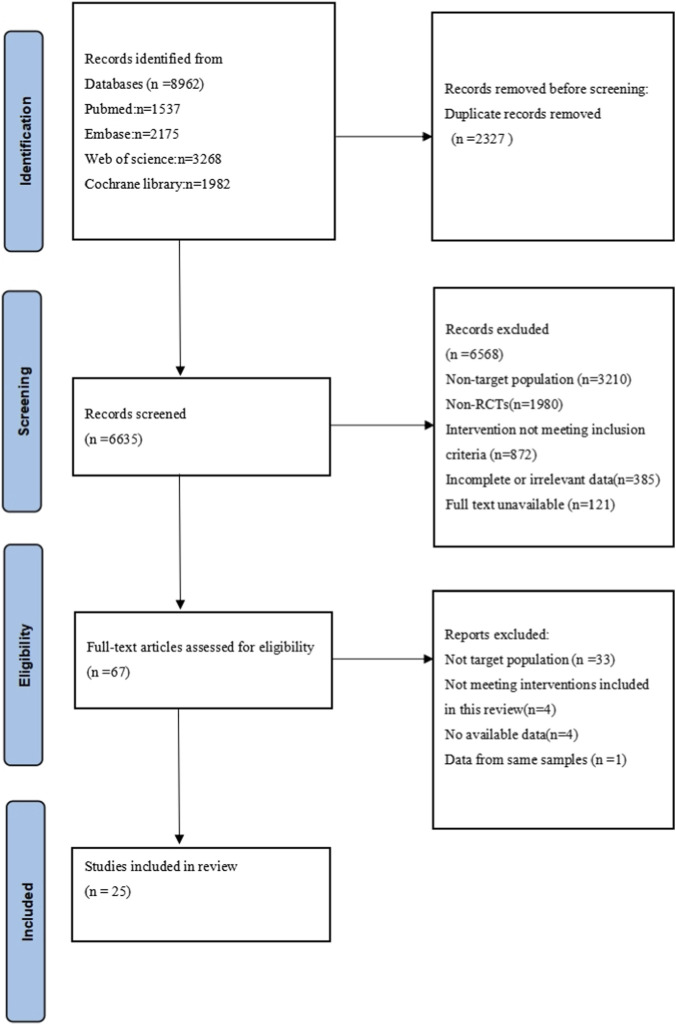
Flow diagram of literature selection.

A total of 8,962 relevant articles were retrieved from electronic databases. Using Endnote, 2,327 duplicate references were removed, leaving 6,635 articles for further review. After screening the titles and abstracts, 6,568 articles were excluded. The remaining 67 articles underwent full-text review, resulting in the exclusion of 41 articles for various reasons, including non-target populations (n = 33), interventions that did not meet the inclusion criteria (n = 4), missing data (n = 4), and duplicated data (n = 1). Consequently, 25 articles meeting the study’s inclusion criteria were included in the final analysis (Bensalem et al., 2019; Bowtell et al., 2017; Calabrese et al., 2008; Carlson et al., 2007; Chai et al., 2019; Cheng et al., 2024; Cox et al., 2015; Cox et al., 2020; Cropley et al., 2012; Crosta et al., 2021; Galduroz and Carlini, 1996; Mcnamara et al., 2018; Mcphee et al., 2021; Miller et al., 2018; Mix and Crews, 2000; Mix and David Crews, 2002; Morgan and Stevens, 2010; Nathan et al., 2002; Perry et al., 2018; Rodrigo-Gonzalo et al., 2023; Scholey et al., 2008; Small et al., 2018; Solomon et al., 2002; Wong et al., 2012; Wood et al., 2023). See Figure 1.

### 3.2 Quality assessment of included studies

**FIGURE 2:**
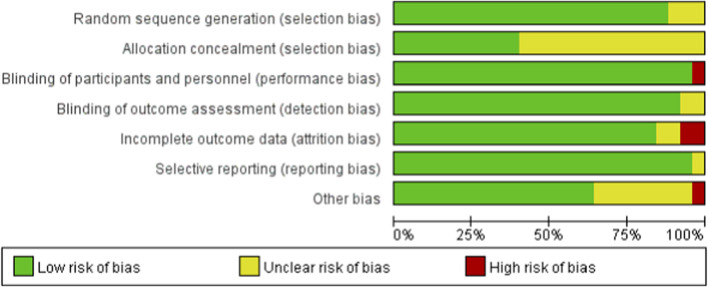
Risk of bias graph for each included study.

This study conducted a rigorous quality assessment of the included studies, with the following results: Regarding random sequence generation, the majority of studies were classified as low risk of bias, with three studies categorized as unclear risk, and none as high risk, indicating that most studies followed appropriate random allocation procedures. In terms of allocation concealment, 15 studies did not specify the measures for allocation concealment, and were therefore assessed as unclear risk. With respect to blinding, only one study, in which the intervention involved raisins, could not implement blinding for participants, resulting in a high risk of bias. For detection bias, two studies lacked specific descriptions of blinding for assessors, and were thus classified as unclear risk. Concerning data completeness, two studies had participant withdrawals from the intervention group (10 and 8 participants, respectively), which could lead to biased results, and were therefore assessed as high risk. Regarding other biases, one study was categorized as high risk due to baseline imbalances between the intervention and control groups, and a small final sample size. In summary, among the 25 studies included, 6 were classified as low risk and 4 as high risk. Although 15 studies did not clearly report the allocation concealment, the description of the random sequence generation process was sufficient, and there were no other indications of high risk. Therefore, the overall assessment is moderate risk. For further details, see Figure 2.

### 3.3 Characteristics of included studies

A total of 25 randomized controlled trials (RCTs) involving 1,861 healthy older adults participants were included in this study. The interventions in the control groups comprised 10 distinct plant active substances and their combinations, as follows: blueberry (n = 5), ginkgo biloba extract (n = 4), bacopa monnieri (n = 3), curcumin (n = 3), caffeine (n = 2), guarana (n = 1), raisins (n = 1), salvia (n = 1), tart cherry (n = 1), wild green oats (n = 1), a combination of grape and blueberry extracts (n = 1), ginkgo leaf + Gotu Kola + DHA (n = 1), bacopa monnieri + lycopene + astaxanthin + vitamin B12 (n = 1), and salvia + Rosmarinus + Melissa (n = 1). Based on the cognitive domains outlined in the Diagnostic and Statistical Manual of Mental Disorders, Fifth Edition (DSM-5) (Sachdev et al., 2014), the outcome measures included: learning and memory function (23 studies), complex attention (18 studies), executive function (16 studies), language function (4 studies), and perceptual motor function (3 studies). The studies originated from various countries: 9 studies from the United States, 6 from Australia, 6 from the United Kingdom, 1 from Brazil, 1 from France, 1 from Italy, and 1 from Spain. The characteristics of the included studies are shown in Table 2. The methods used for the extraction of plant active substances are detailed in Supplementary Table S5.

**TABLE 2:** Characteristics of the studies included in the meta-analysis.

Author	Country	Year	Age (mean + sd)	Total/Male/Female	Interventions	Dosage	Treatment duration	Control	Outcomes
J. C. Galduróz	Brazil	1996	I + C:65.6(NA)	I:15/NA/NAI:15/NA/NAC:15/NA/NA	Caffeine/Guarana	25 mg/1000 mg/d	5 months	Placebo	SDMT, DS, mosaic test
J. A. Mix	United States	2000	I:67.50 (9.23) C:68.65 (6.95)	I:20/NA/NAC:20/NA/NA	Ginkgo biloba	180 mg/d	6 weeks	Placebo	TMT-A, WMS-R, SCWT
Joseph A. Mix	United States	2002	I:66.97 (6.12) C:68.60 (6.96)	I:127/NA/NAC:122/NA/NA	Ginkgo biloba	180 mg/d	6 weeks	Placebo	WAIS-Ⅲ, SRT
P. J. Nathan	Australia	2002	I:58.46 (10.92) C:58.46 (10/92)	I:11/6/5 C:11/6/5	Ginkgo biloba	120 mg/d	90 min	Placebo	CDR (Choice reaction time; Spatial working memory), AVLT
P. R. Solomon	United States	2002	I:68.7 (4.7) C:69.9 (5.4)	I:111/46/65 C:108/45/63	Ginkgo biloba	120 mg/d	6 weeks	Placebo	SDMT, WMS-R, SCWT, BNT
J. J. Carlson	United States	2007	I:73.1 (4.8) C:72.1 (6.0)	I:42/21/21 C:36/21/15	Ginkgo biloba + Gotu kola + DHA	160 mg + 68 mg + 180 mg/d	4 months	Placebo	SDMT, list learning, COWAT, JLO
C. Calabrese	United States	2008	I + C:73.5(NA)	I:24/NA/NAC:24/NA/NA	Bacopa monnieri	300 mg/d	12 weeks	Placebo	AVLT, SCWT
A. B. Scholey	United Kingdom	2008	I:72.9(NA) C:72.9(NA)	I:20/11/9 C:20/11/9	Salvia	333 mg/d	4 h	Placebo	CDR (Choice reaction time; Spatial working memory; delayed word recall)
A. Morgan	Australia	2010	I:65.41 (6.87) C:65.39 (8.20)	I:49/24/25 C:49/28/21	Bacopa monnieri	300 mg/d	12 weeks	Placebo	TMT-A, AVLT, TMT-B
V. Cropley	Australia	2012	I:62.5 (6.0) C:62.5 (6.0)	I:39/20/19 C:39/20/19	Caffeine	167 mg/d	40 min	Placebo	RVIP, VVLT, SCWT
R. H. X. Wong	Australia	2012	I:67 (0.8) C:67 (0.8)	I:37/12/25 C:37/12/25	Wild green oat	1500 mg/d	12 weeks	Placebo	TMT-A, TMT-B
K. H. Cox	Australia	2015	I:67.56 (4.479) C:69.43 (6.579)	I:30/12/18 C:30/10/20	Curcumin	80 mg/d	4 weeks	Placebo	Serials subtraction task
J. L. Bowtell	United Kingdom	2017	I:67.5 (0.9) C:69.9 (0.9)	I:12/7/5 C:14/6/8	Blueberry	30 mL/d	12 weeks	Placebo	GMLT, ISLT,Identification task
R. K. McNamara	United States	2017	I:68 (3.9) C:67 (4.9)	I:19/8/11 C:20/10/10	Blueberry	25 g/d	24 weeks	Placebo	HVLT
M. G. Miller	United States	2017	I:67.8 (4.6) C:67.3 (4.8)	I:18/5/13 C:19/7/12	Blueberry	24 g/d	90 days	Placebo	CVLT
N. S. L. Perry	United Kingdom	2018	I + C:61 (9.26)	I:12/NA/NAC:6/NA/NA	Salvia + Rosmarinus + Melissa	10 mL/d	2 weeks	Placebo	Delayed word Recall
G. W. Small	United States	2018	I:63.1 (8.4) C:62.9 (9.4)	I:21/9/12 C:19/9/10	Curcumin	180 mg/d	18 months	Placebo	TMT-A, SRT
J. Bensalem	France	2019	I + C:64.66 (2.91)	I:91/NA/NAC:98/NA/NA	Grape + Blueberry	600 mg/d	24 weeks	Placebo	CANTAB(Spatial Span; VRMFR)
S. C. Chai	United States	2019	I:70.0 (3.7) C:69.5 (3.9)	I:20/8/12 C:17/9/8	Tart cherry juice	480 mL/d	12 weeks	Placebo	CANTAB (reaction time; Spatial working memory; PAL)
K. H. M. Cox	Australia	2020	I:67.81 (6.00) C:68.38 (6.71)	I:42/21/21 C:43/21/22	Curcumin	80 mg/d	12 weeks	Placebo	vMWM, AFT,serials subtraction task
F. Crosta	Italy	2021	I:61.88 (1.36) C:62.05 (1.55)	I:40/12/28 C:40/13/27	Bacopa + Lycopene + Astaxanthin + Vitamin B12	NA	8 weeks	Placebo	TMT-A, AVLT, TMT-B, VFT
G. M. McPhee	United Kingdom	2021	I:68.87 (5.59) C:68.85 (5.93)	I:15/7/8 C:13/5/8	Bacopa monnieri	4.32 g/d	12 weeks	Placebo	Choice reaction time, delayed word recall, numeric working memory
M. J. Rodrigo-Gonzalo	Spain	2023	I:76.3 (4.6) C:77.0 (4.6)	I:40/10/30 C:40/17/23	Raisin	50 g/d	6 months	Placebo	AVLT, category fluency
E. Wood	United Kingdom	2023	I:69.44 (3.48) C:70.76 (3.81)	I:32/12/20 C:29/12/17	Blueberry	26 g/d	12 weeks	Placebo	AVLT, TST, serials subtraction task
N. Cheng	United Kingdom	2024	I + C:71.02 (2.03)	I:45/18/27 C:45/18/27	Blueberry	200 mg/d	2 h	Placebo	AVLT, TST

Definition of cognitive domais and assessments: The cognitive outcomes in this study were defined and classified according to the core cognitive domains outlined in the Diagnostic and Statistical Manual of Mental Disorders (Fifth Edition) (DSM-5). The representative neuropsychological tests included in each domain are as follows:①Learning and Memory: AVLT, HVLT, RAVLT, SRT, WMS-R, CVLT, VVLT, vMWM, list learning, Delayed Word Recall, CANTAB(VRMFR; PAL).②Complex Attention: SDMT, TMT-A, RVIP, WAIS-III-Digit Symbol, Serials Subtraction Task, Identification task, CDR (Choice Reaction Time), CANTAB (Reaction Time).③Executive Function: SCWT, TMT-B, GMLT, spatial working memory, Numeric working memory, AFT, TST, CDR (Spatial Working Memory), CANTAB (Spatial Working Memory; Spatial Span).④Language: BNT, COWAT, VFT, Category Fluency.⑤Perceptual-Motor: JLO, mosaic test, WAIS-R-BD.

Abbreviations: AVLT: auditory verbal learning test; BNT: boston naming test; CANTAB:cambridge neuropsychological test automated battery; CDR: cognitive drug research computerised assessment system; COWAT: controlled oral word association test; CVLT: california verbal learning test; DS: digital span; GMLT:groton maze learning test; HVLT: hopkins verbal learning test; ISLT: international shopping list task; JLO:judgment of line orientation test; PAL:Paired Associates Learning, RVIP:rapid visual information processing; SRT: selective reminding test; SCWT: stroop color and word test; SDMT: symbol digit modalities test; TMT-A: Trail Making Test-A; TMT-B: Trail Making Test-B; TST: Task-switching test; vMWM:virtual morris water maze; VVLT:visual verbal learning test; VFT:verbal fluency test; VRMFR: Visual Recognition Memory-Free Recall; WAIS-Ⅲ:Wechsler Adult Intelligence Scale Ⅲ; WMS-R:Wechsler Memory Scale-Revised.

### 3.4 Network meta-analysis

**FIGURE 3:**
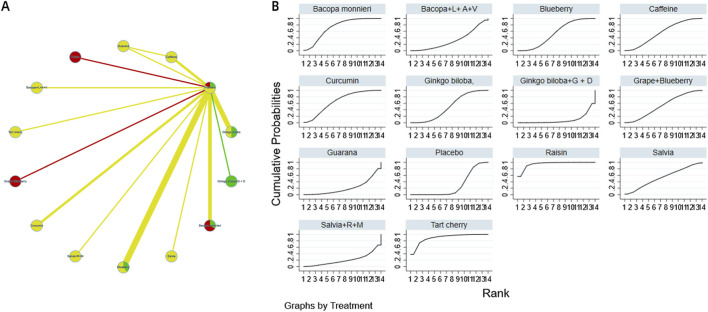
NMA and SUCRA plot for learning and memory. Note: **(A)**, network graph of the comparisons for Learning and Memory. **(B)**, the SUCRA plot for Learning and Memory. The thickness of the connecting lines is proportional to the number of trials for that direct comparison. The color of the lines represents the overall risk of bias for the direct comparison: red for high risk, yellow for moderate/unclear risk, and green for low risk. Abbreviations: Bacopa + L + A + V: Bacopa + Lycopene + Astaxanthin + Vitamin B12; Ginkgo biloba + G + D: Ginkgo biloba + Gotu kola + DHA; Salvia + R + M: Salvia + Rosmarinus + Melissa.

**FIGURE 4:**
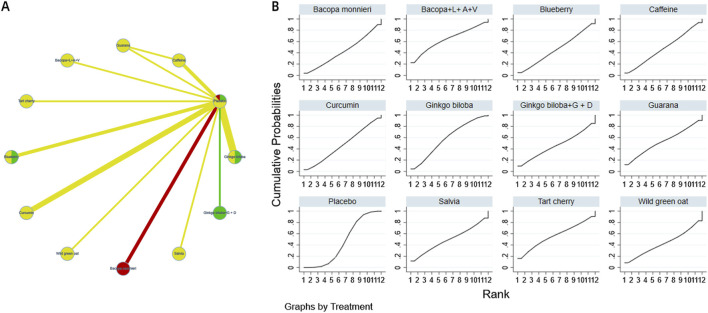
NMA and SUCRA plot for complex attention. Note: **(A)**, network graph of the comparisons for Complex Attention. **(B)**, the SUCRA plot for Complex Attention. The thickness of the connecting lines is proportional to the number of trials for that direct comparison. The color of the lines represents the overall risk of bias for the direct comparison: red for high risk, yellow for moderate/unclear risk, and green for low risk. Abbreviations: Bacopa + L + A + V: Bacopa + Lycopene + Astaxanthin + Vitamin B12; Ginkgo biloba + G + D: Ginkgo biloba + Gotu kola + DHA.

**FIGURE 5:**
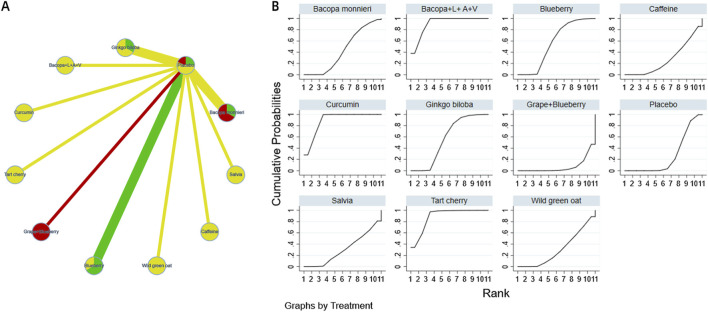
NMA and SUCRA plot for executive function. Note: **(A)**, network graph of the comparisons for Executive Function. **(B)**, the SUCRA plot for Executive Function. The thickness of the connecting lines is proportional to the number of trials for that direct comparison. The color of the lines represents the overall risk of bias for the direct comparison: red for high risk, yellow for moderate/unclear risk, and green for low risk. Abbreviations: Bacopa + L + A + V: Bacopa + Lycopene + Astaxanthin + Vitamin B12.

**FIGURE 6:**
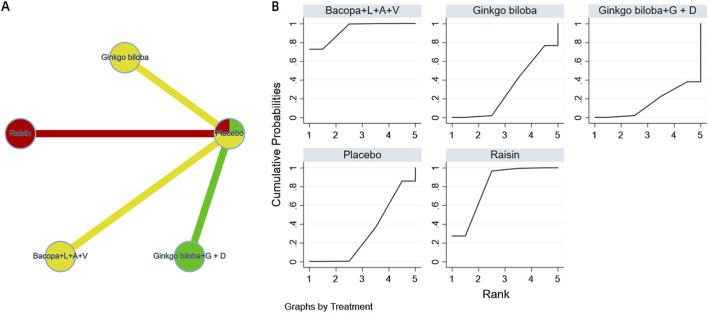
NMA and SUCRA plot for language. Note: **(A)**, network graph of the comparisons for Language. **(B)**, the SUCRA plot for Language. The thickness of the connecting lines is proportional to the number of trials for that direct comparison. The color of the lines represents the overall risk of bias for the direct comparison: red for high risk, yellow for moderate/unclear risk, and green for low risk. Abbreviations:Bacopa + L + A + V: Bacopa + Lycopene + Astaxanthin + Vitamin B12; Ginkgo biloba + G + D: Ginkgo biloba + Gotu kola + DHA.

**FIGURE 7:**
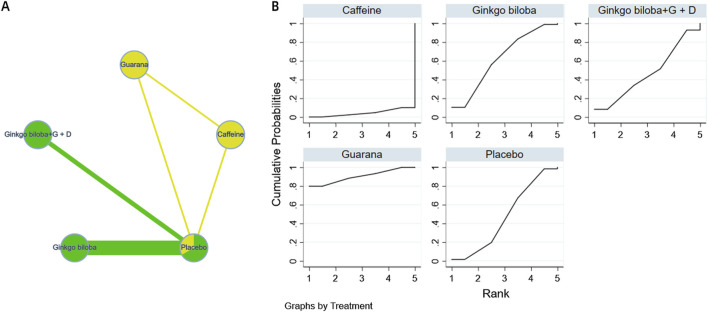
NMA and SUCRA plot for perceptual-motor function. Note: **(A)**, network graph of the comparisons for Perceptual-Motor Function. **(B)**, the SUCRA plot for Perceptual-Motor Function. The thickness of the connecting lines is proportional to the number of trials for that direct comparison. The color of the lines represents the overall risk of bias for the direct comparison: red for high risk, yellow for moderate/unclear risk, and green for low risk. Abbreviations: Ginkgo biloba + G + D: Ginkgo biloba + Gotu kola + DHA.

The complete network meta-analysis (NMA) diagrams are shown in Figures 3A, 4A, 5A, 6A, 7A. In these diagrams, the thickness of the lines reflects the number of studies, while the color of the nodes and lines indicates the risk of bias, categorized into high, low, and unclear risk proportions. A summary of the CINeMA evidence grading results is presented in Supplementary Table S6.

#### 3.4.1 Learning and memory function

Consistency and inconsistency tests were performed for all indirect and direct comparisons across the studies, with p-values greater than 0.05, indicating that the consistency effect among the studies is acceptable. Further details are provided in Supplementary Table S7.

The results of the network meta-analysis demonstrate that, compared to the placebo group, raisins (Raisin) [MD = 1.09, 95% CI=(0.46,1.71)], tart cherry (Tart cherry) [MD = 0.96, 95% CI=(0.13,1.78)], and Bacopa monnieri extract (Bacopa monnieri) [MD = 0.49, 95% CI=(0.07,0.90)] show significant advantages in improving memory function. The detailed results are presented in Table 3.

**TABLE 3:** League table on learning and memory.

Raisin	Tart cherry	Bacopa monnieri	Curcumin	Caffeine	Blueberry	Salvia	Grape + Blueberry	Ginkgo biloba	Bacopa + L + A + V	Placebo	Guarana	Salvia + R + M	Ginkgo biloba + G + D
Raisin	−0.13 (−1.16,0.90)	−0.60 (−1.35,0.15)	−0.68 (−1.46,0.11)	−0.80 (−1.59,-0.01)	−0.84 (−1.54,-0.14)	−0.83 (−1.80,0.15)	−0.83 (−1.63,-0.03)	−0.88 (−1.57,-0.19)	−1.11 (−1.97,-0.24)	−1.09 (−1.71,-0.46)	−1.31 (−2.29,-0.33)	−1.42 (−2.66,-0.18)	−1.55 (−2.42,-0.68)
0.13 (−0.90,1.16)	Tart cherry	−0.47 (−1.39,0.45)	−0.55 (−1.50,0.40)	−0.67 (−1.63,0.29)	−0.71 (−1.59,0.17)	−0.70 (−1.81,0.42)	−0.70 (−1.67,0.26)	−0.75 (−1.63,0.13)	−0.97 (−2.00,0.05)	−0.96 (−1.78,-0.13)	−1.18 (−2.30,-0.06)	−1.29 (−2.64,0.06)	−1.42 (−2.44,-0.39)
0.60 (−0.15,1.35)	0.47 (−0.45,1.39)	Bacopa monnieri	−0.08 (−0.71,0.56)	−0.20 (−0.84,0.44)	−0.24 (−0.77,0.29)	−0.23 (−1.08,0.63)	−0.23 (−0.88,0.42)	−0.28 (−0.80,0.24)	−0.50 (−1.24,0.23)	−0.49 (−0.90,-0.07)	−0.71 (−1.58,0.15)	−0.82 (−1.96,0.33)	−0.95 (−1.69,-0.21)
0.68 (−0.11,1.46)	0.55 (−0.40,1.50)	0.08 (−0.56,0.71)	Curcumin	−0.12 (−0.80,0.56)	−0.16 (−0.73,0.41)	−0.15 (−1.03,0.73)	−0.16 (−0.84,0.53)	−0.20 (−0.76,0.35)	−0.43 (−1.19,0.34)	−0.41 (−0.88,0.06)	−0.63 (−1.53,0.26)	−0.74 (−1.91,0.43)	−0.87 (−1.64,-0.10)
**0.80 (0.01,1.59)**	0.67 (−0.29,1.63)	0.20 (−0.44,0.84)	0.12 (−0.56,0.80)	Caffeine	−0.04 (−0.62,0.54)	−0.03 (−0.92,0.87)	−0.03 (−0.73,0.66)	−0.08 (−0.65,0.49)	−0.30 (−1.08,0.47)	−0.29 (−0.78,0.20)	−0.51 (−1.27,0.25)	−0.62 (−1.79,0.56)	−0.75 (−1.53,0.03)
**0.84 (0.14,1.54)**	0.71 (−0.17,1.59)	0.24 (−0.29,0.77)	0.16 (−0.41,0.73)	0.04 (−0.54,0.62)	Blueberry	0.01 (−0.80,0.82)	0.01 (−0.59,0.60)	−0.04 (−0.48,0.39)	−0.27 (−0.95,0.42)	−0.25 (−0.57,0.07)	−0.47 (−1.29,0.35)	−0.58 (−1.69,0.54)	−0.71 (−1.40,-0.02)
0.83 (−0.15,1.80)	0.70 (−0.42,1.81)	0.23 (−0.63,1.08)	0.15 (−0.73,1.03)	0.03 (−0.87,0.92)	−0.01 (−0.82,0.80)	Salvia	−0.01 (−0.90,0.89)	−0.06 (−0.86,0.75)	−0.28 (−1.24,0.68)	−0.26 (−1.01,0.48)	−0.49 (−1.55,0.58)	−0.59 (−1.89,0.71)	−0.72 (−1.68,0.24)
**0.83 (0.03,1.63)**	0.70 (−0.26,1.67)	0.23 (−0.42,0.88)	0.16 (−0.53,0.84)	0.03 (−0.66,0.73)	−0.01 (−0.60,0.59)	0.01 (−0.89,0.90)	Grape + Blueberry	−0.05 (−0.63,0.53)	−0.27 (−1.05,0.51)	−0.25 (−0.75,0.25)	−0.48 (−1.39,0.43)	−0.58 (−1.76,0.60)	−0.71 (−1.50,0.07)
**0.88 (0.19,1.57)**	0.75 (−0.13,1.63)	0.28 (−0.24,0.80)	0.20 (−0.35,0.76)	0.08 (−0.49,0.65)	0.04 (−0.39,0.48)	0.06 (−0.75,0.86)	0.05 (−0.53,0.63)	Ginkgo biloba	−0.22 (−0.90,0.45)	−0.21 (−0.50,0.09)	−0.43 (−1.24,0.38)	−0.54 (−1.65,0.57)	−0.67 (−1.34,0.01)
**1.11 (0.24,1.97)**	0.97 (−0.05,2.00)	0.50 (−0.23,1.24)	0.43 (−0.34,1.19)	0.30 (−0.47,1.08)	0.27 (−0.42,0.95)	0.28 (−0.68,1.24)	0.27 (−0.51,1.05)	0.22 (−0.45,0.90)	Bacopa + L + A + V	0.02 (−0.59,0.62)	−0.21 (−1.18,0.76)	−0.31 (−1.54,0.92)	−0.44 (−1.30,0.41)
**1.09 (0.46,1.71)**	**0.96 (0.13,1.78)**	**0.49 (0.07,0.90)**	0.41 (−0.06,0.88)	0.29 (−0.20,0.78)	0.25 (−0.07,0.57)	0.26 (−0.48,1.01)	0.25 (−0.25,0.75)	0.21 (−0.09,0.50)	−0.02 (−0.62,0.59)	Placebo	−0.22 (−0.98,0.53)	−0.33 (−1.40,0.74)	−0.46 (−1.07,0.15)
**1.31 (0.33,2.29)**	**1.18 (0.06,2.30)**	0.71 (−0.15,1.58)	0.63 (−0.26,1.53)	0.51 (−0.25,1.27)	0.47 (−0.35,1.29)	0.49 (−0.58,1.55)	0.48 (−0.43,1.39)	0.43 (−0.38,1.24)	0.21 (−0.76,1.18)	0.22 (−0.53,0.98)	Guarana	−0.11 (−1.42,1.20)	−0.24 (−1.21,0.74)
**1.42 (0.18,2.66)**	1.29 (−0.06,2.64)	0.82 (−0.33,1.96)	0.74 (−0.43,1.91)	0.62 (−0.56,1.79)	0.58 (−0.54,1.69)	0.59 (−0.71,1.89)	0.58 (−0.60,1.76)	0.54 (−0.57,1.65)	0.31 (−0.92,1.54)	0.33 (−0.74,1.40)	0.11 (−1.20,1.42)	Salvia + R + M	−0.13 (−1.36,1.10)
**1.55 (0.68,2.42)**	**1.42 (0.39,2.44)**	**0.95 (0.21,1.69)**	**0.87 (0.10,1.64)**	0.75 (−0.03,1.53)	**0.71 (0.02,1.40)**	0.72 (−0.24,1.68)	0.71 (−0.07,1.50)	0.67 (−0.01,1.34)	0.44 (−0.41,1.30)	0.46 (−0.15,1.07)	0.24 (−0.74,1.21)	0.13 (−1.10,1.36)	Ginkgo biloba + G + D

Data are reported as MD, with 95% CI., statistically significant differences are highlighted in bold.

Abbreviations:Bacopa + L + A + V:Bacopa + Lycopene + Astaxanthin + Vitamin B12; Ginkgo biloba + G + D:Ginkgo biloba + Gotu kola + DHA; Salvia + R + M:Salvia + Rosmarinus + Melissa.

Statistically significant differences are highlighted in bold (p < 0.05).

The probability ranking of different plant active substances for improving memory function indicates that raisin (SUCRA: 95.1%) and tart cherry (SUCRA: 89.5%) are most likely to be the optimal interventions. However, this probability ranking should be interpreted with caution in conjunction with the effect sizes and their confidence intervals reported in Table 3. For instance, although raisin extract and tart cherry occupied the top two positions in the ranking, the direct comparison between them showed no statistically significant difference [MD = 0.13, 95% CI=(-0.90,1.16)]. This suggests that while SUCRA values provide a probabilistic estimate of the hierarchy of interventions, the actual efficacy differences between closely ranked interventions may not be statistically significant. The details are shown in Figure 3B.

#### 3.4.2 Complex attention function

Consistency and inconsistency tests were conducted for all indirect and direct comparisons across the studies, with p-values greater than 0.05, indicating that the consistency effect among the studies is acceptable. Further details are provided in Supplementary Table S8.

The results of the network meta-analysis indicate that most plant active substances show no statistically significant differences compared to the placebo in improving attention function. The details are provided in Table 4.

**TABLE 4:** League table on complex attention.

Guarana	Salvia	Caffeine	Curcumin	Blueberry	Ginkgo biloba + G + D	Placebo	Wild green oat	Bacopa monnieri
−0.53 (−4.88,3.83)	−0.64 (−5.13,3.85)	−0.75 (−4.63,3.13)	−0.75 (−4.42,2.91)	−0.82 (−4.71,3.07)	−0.85 (−5.32,3.62)	−0.80 (−3.96,2.37)	−0.97 (−5.44,3.50)	−0.95 (−4.83,2.93)
−0.16 (−3.56,3.23)	−0.28 (−3.84,3.29)	−0.38 (−3.14,2.37)	−0.39 (−2.84,2.06)	−0.46 (−3.22,2.31)	−0.49 (−4.02,3.05)	−0.43 (−2.02,1.16)	−0.60 (−4.14,2.94)	−0.58 (−3.34,2.18)
−0.13 (−4.52,4.25)	−0.25 (−4.76,4.27)	−0.35 (−4.27,3.56)	−0.36 (−4.06,3.34)	−0.43 (−4.35,3.49)	−0.46 (−4.95,4.04)	−0.40 (−3.60,2.80)	−0.57 (−5.07,3.92)	−0.55 (−4.47,3.36)
Guarana	−0.11 (−4.49,4.26)	−0.22 (−3.22,2.78)	−0.23 (−3.75,3.30)	−0.29 (−4.05,3.46)	−0.32 (−4.68,4.03)	−0.27 (−3.27,2.73)	−0.44 (−4.80,3.92)	−0.42 (−4.17,3.33)
0.11 (−4.26,4.49)	Salvia	−0.11 (−4.01,3.80)	−0.11 (−3.80,3.58)	−0.18 (−4.09,3.73)	−0.21 (−4.70,4.28)	−0.15 (−3.34,3.03)	−0.33 (−4.82,4.16)	−0.31 (−4.21,3.60)
0.22 (−2.78,3.22)	0.11 (−3.80,4.01)	Caffeine	−0.01 (−2.93,2.91)	−0.07 (−3.26,3.12)	−0.10 (−3.98,3.78)	−0.05 (−2.30,2.20)	−0.22 (−4.10,3.66)	−0.20 (−3.39,2.98)
0.23 (−3.30,3.75)	0.11 (−3.58,3.80)	0.01 (−2.91,2.93)	Curcumin	−0.07 (−3.00,2.86)	−0.10 (−3.76,3.57)	−0.04 (−1.90,1.82)	−0.22 (−3.88,3.45)	−0.20 (−3.12,2.73)
0.29 (−3.46,4.05)	0.18 (−3.73,4.09)	0.07 (−3.12,3.26)	0.07 (−2.86,3.00)	Blueberry	−0.03 (−3.92,3.85)	0.03 (−2.24,2.29)	−0.15 (−4.04,3.74)	−0.13 (−3.32,3.07)
0.32 (−4.03,4.68)	0.21 (−4.28,4.70)	0.10 (−3.78,3.98)	0.10 (−3.57,3.76)	0.03 (−3.85,3.92)	Ginkgo biloba + G + D	0.06 (−3.10,3.22)	−0.12 (−4.59,4.35)	−0.10 (−3.98,3.78)
0.27 (−2.73,3.27)	0.15 (−3.03,3.34)	0.05 (−2.20,2.30)	0.04 (−1.82,1.90)	−0.03 (−2.29,2.24)	−0.06 (−3.22,3.10)	Placebo	−0.17 (−3.33,2.99)	−0.15 (−2.41,2.10)
0.44 (−3.92,4.80)	0.33 (−4.16,4.82)	0.22 (−3.66,4.10)	0.22 (−3.45,3.88)	0.15 (−3.74,4.04)	0.12 (−4.35,4.59)	0.17 (−2.99,3.33)	Wild green oat	0.02 (−3.86,3.90)
0.42 (−3.33,4.17)	0.31 (−3.60,4.21)	0.20 (−2.98,3.39)	0.20 (−2.73,3.12)	0.13 (−3.07,3.32)	0.10 (−3.78,3.98)	0.15 (−2.10,2.41)	−0.02 (−3.90,3.86)	Bacopa monnieri

Data are reported as MD, with 95% CI., statistically significant differences are highlighted in bold.

Abbreviations: Bacopa + L + A + V:Bacopa + Lycopene + Astaxanthin + Vitamin B12; Ginkgo biloba + G + D:Ginkgo biloba + Gotu kola + DHA.

The probability ranking of different plant active substances in improving attention shows that the Bacopa monnieri compound (Bacopa + L + A + V, Bacopa + Lycopene + Astaxanthin + Vitamin B12) ranks first (SUCRA:63.9%). However, the SUCRA values of each intervention measure are relatively concentrated, and the confidence intervals of their effect sizes overlap widely, indicating that the differences in rankings lack statistical significance support. Therefore, the differences in the effects of different intervention measures on improving complex attention may not have clinical significance and further research is needed for verification. The details are shown in Figure 4B.

#### 3.4.3 Executive function

Consistency and inconsistency tests were performed for both indirect and direct comparisons across all studies, with p-values greater than 0.05, indicating that the consistency effect among the studies is acceptable. Further details are provided in Supplementary Table S9.

The results of the network meta-analysis indicate that, compared to the placebo group, Bacopa monnieri compound [MD = 1.28, 95% CI = (0.78, 1.78)], curcumin [MD = 1.12, 95% CI = (0.72, 1.70)], tart cherry [MD = 1.21, 95% CI = (0.47, 1.96)], and Ginkgo biloba extract [MD = 0.30, 95% CI = (0.01, 0.59)] show significant advantages in improving executive function. The details are shown in Table 5.

**TABLE 5:** League table on executive function.

Bacopa + L + A + V	Curcumin	Tart cherry	Ginkgo biloba	Blueberry	Bacopa monnieri	Salvia	Wild green oat	Caffeine	Placebo	Grape + Blueberry
Bacopa + L + A + V	−0.06 (−0.77,0.64)	−0.06 (−0.96,0.84)	−0.98 (−1.56,-0.40)	−0.99 (−1.58,-0.40)	−1.12 (−1.74,-0.50)	−1.27 (−2.07,-0.46)	−1.25 (−1.94,-0.57)	−1.29 (−1.97,-0.62)	−1.28 (−1.78,-0.78)	−1.52 (−2.11,-0.94)
0.06 (−0.64,0.77)	Curcumin	0.00 (−0.89,0.90)	−0.92 (−1.49,-0.34)	−0.92 (−1.51,-0.34)	−1.06 (−1.67,-0.44)	−1.20 (−2.00,-0.40)	−1.19 (−1.87,-0.51)	−1.23 (−1.90,-0.56)	−1.21 (−1.70,-0.72)	−1.46 (−2.04,-0.88)
0.06 (−0.84,0.96)	−0.00 (−0.90,0.89)	Tart cherry	−0.92 (−1.72,-0.12)	−0.93 (−1.74,-0.12)	−1.06 (−1.89,-0.23)	−1.20 (−2.18,-0.23)	−1.19 (−2.07,-0.31)	−1.23 (−2.11,-0.36)	−1.21 (−1.96,-0.47)	−1.46 (−2.27,-0.65)
**0.98 (0.40,1.56)**	**0.92 (0.34,1.49)**	**0.92 (0.12,1.72)**	Ginkgo biloba	−0.01 (−0.42,0.41)	−0.14 (−0.55,0.27)	−0.29 (−0.98,0.41)	−0.27 (−0.82,0.28)	−0.31 (−0.85,0.23)	−0.30 (−0.59,-0.01)	−0.54 (−0.96,-0.12)
**0.99 (0.40,1.58)**	**0.92 (0.34,1.51)**	**0.93 (0.12,1.74)**	0.01 (−0.41,0.42)	Blueberry	−0.13 (−0.60,0.33)	−0.28 (−0.98,0.42)	−0.26 (−0.82,0.30)	−0.31 (−0.86,0.25)	−0.29 (−0.60,0.02)	−0.53 (−0.97,-0.10)
**1.12 (0.50,1.74)**	**1.06 (0.44,1.67)**	**1.06 (0.23,1.89)**	0.14 (−0.27,0.55)	0.13 (−0.33,0.60)	Bacopa monnieri	−0.14 (−0.87,0.58)	−0.13 (−0.72,0.46)	−0.17 (−0.75,0.41)	−0.16 (−0.52,0.21)	−0.40 (−0.87,0.07)
**1.27 (0.46,2.07)**	**1.20 (0.40,2.00)**	**1.20 (0.23,2.18)**	0.29 (−0.41,0.98)	0.28 (−0.42,0.98)	0.14 (−0.58,0.87)	Salvia	0.01 (−0.77,0.80)	−0.03 (−0.80,0.75)	−0.01 (−0.64,0.62)	−0.26 (−0.95,0.44)
**1.25 (0.57,1.94)**	**1.19 (0.51,1.87)**	**1.19 (0.31,2.07)**	0.27 (−0.28,0.82)	0.26 (−0.30,0.82)	0.13 (−0.46,0.72)	−0.01 (−0.80,0.77)	Wild green oat	−0.04 (−0.69,0.61)	−0.03 (−0.49,0.44)	−0.27 (−0.83,0.29)
**1.29 (0.62,1.97)**	**1.23 (0.56,1.90)**	**1.23 (0.36,2.11)**	0.31 (−0.23,0.85)	0.31 (−0.25,0.86)	0.17 (−0.41,0.75)	0.03 (−0.75,0.80)	0.04 (−0.61,0.69)	Caffeine	0.02 (−0.44,0.47)	−0.23 (−0.77,0.32)
**1.28 (0.78,1.78)**	**1.21 (0.72,1.70)**	**1.21 (0.47,1.96)**	**0.30 (0.01,0.59)**	0.29 (−0.02,0.60)	0.16 (−0.21,0.52)	0.01 (−0.62,0.64)	0.03 (−0.44,0.49)	−0.02 (−0.47,0.44)	Placebo	−0.24 (−0.55,0.06)
**1.52 (0.94,2.11)**	**1.46 (0.88,2.04)**	**1.46 (0.65,2.27)**	**0.54 (0.12,0.96)**	**0.53 (0.10,0.97)**	0.40 (−0.07,0.87)	0.26 (−0.44,0.95)	0.27 (−0.29,0.83)	0.23 (−0.32,0.77)	0.24 (−0.06,0.55)	Grape + Blueberry

Data are reported as MD, with 95% CI., statistically significant differences are highlighted in bold.

Abbreviations: Bacopa + L + A + V:Bacopa + Lycopene + Astaxanthin + Vitamin B12.

Statistically significant differences are highlighted in bold (p < 0.05).

The probability ranking of different plant active substances in improving executive function shows that the Bacopa monnieri compound (SUCRA:91.3%), curcumin (SUCRA:89.3%), and tart cherry (SUCRA:88.9%) rank in the top three. Although the SUCRA probability is higher, the confidence intervals of its effect sizes still partially overlap. The ranking should be regarded as a probabilistic indication rather than a definitive conclusion. The details are shown in Figure 5B.

#### 3.4.4 Language function

The network meta-analysis results indicate that, compared to the placebo group, Bacopa monnieri complex [MD = 0.75, 95% CI = (0.29, 1.21)] and raisins [MD = 0.50, 95% CI = (0.29, 1.21)] show significant advantages in improving language function (see Table 6).

**TABLE 6:** League table on language.

Bacopa + L + A + V	Raisin	Placebo	Ginkgo biloba	Ginkgo biloba + G + D
A = Bacopa + L + A + V	−0.20 (−0.84,0.44)	−0.75 (−1.21,-0.29)	−0.75 (−1.29,-0.21)	−0.88 (−1.52,-0.24)
0.20 (−0.44,0.84)	Raisin	−0.55 (−1.00,-0.10)	−0.55 (−1.08,-0.03)	−0.68 (−1.32,-0.05)
**0.75 (0.29,1.21)**	**0.55 (0.10,1.00)**	Placebo	0.00 (−0.28,0.28)	−0.13 (−0.58,0.31)
**0.75 (0.21,1.29)**	**0.55 (0.03,1.08)**	−0.00 (−0.28,0.28)	Ginkgo biloba	−0.13 (−0.66,0.39)
**0.88 (0.24,1.52)**	**0.68 (0.05,1.32)**	0.13 (−0.31,0.58)	0.13 (−0.39,0.66)	Ginkgo biloba + G + D

Data are reported as MD, with 95% CI., statistically significant differences are highlighted in bold.

Abbreviations:Bacopa + L + A + V:Bacopa + Lycopene + Astaxanthin + Vitamin B12; Ginkgo biloba + G + D:Ginkgo biloba + Gotu kola + DHA.

Statistically significant differences are highlighted in bold (p < 0.05).

The probability ranking of different plant active substances in improving language function reveals that Bacopa monnieri compound ranks first (SUCRA: 93%), followed by raisin extract (SUCRA: 80.7%). However, because only four studies were included in the domain of language function, the evidence base is limited; therefore, these rankings should be interpreted with caution and require further verification in larger-sample studies. The details are shown in Figure 6B.

#### 3.4.5 Perceptual-motor

The network meta-analysis results show that guarana [MD = 1.09, 95% CI = (0.349, 1.85)] does not show significant statistical differences when compared to other interventions (such as Ginkgo biloba extract, placebo, and composite Ginkgo formulations) but is significantly superior to caffeine (see Table 7).

**TABLE 7:** League table on perceptual-motor function.

Guarana	Ginkgo biloba	Placebo	Ginkgo biloba + G + D	Caffeine
Guarana	−0.39 (−1.16,0.37)	−0.47 (−1.19,0.25)	−0.49 (−1.34,0.35)	−1.09 (−1.85,-0.34)
0.39 (−0.37,1.16)	Ginkgo biloba	−0.08 (−0.32,0.17)	−0.10 (−0.61,0.41)	−0.70 (−1.47,0.07)
0.47 (−0.25,1.19)	0.08 (−0.17,0.32)	Placebo	−0.03 (−0.47,0.42)	−0.62 (−1.35,0.11)
0.49 (−0.35,1.34)	0.10 (−0.41,0.61)	0.03 (−0.42,0.47)	Ginkgo biloba + G + D	−0.60 (−1.45,0.26)
**1.09 (0.34,1.85)**	0.70 (−0.07,1.47)	0.62 (−0.11,1.35)	0.60 (−0.26,1.45)	Caffeine

Data are reported as MD, with 95% CI., statistically significant differences are highlighted in bold.

Abbreviations: Ginkgo biloba + G + D:Ginkgo biloba + Gotu kola + DHA.

Statistically significant differences are highlighted in bold (p < 0.05).

The probability ranking of different plant active substances in improving perceptual motor function shows that guarana (SUCRA = 90.3%) significantly outperforms other interventions, followed by Ginkgo biloba extract (SUCRA = 62.1%) (see Figure 7B). However, it must be emphasized that this network comprised only three studies and was sparsely connected, resulting in an extremely low strength of evidence. Therefore, the ranking should be regarded as an exploratory finding that urgently warrants confirmation from more high-quality trials. The details are shown in Figure 7B.

### 3.5 Adverse events

Among the 25 RCTs included, 8 clearly recorded adverse events (see Table 8), while the others either did not report or merely stated “no obvious adverse reactions”. The reported events were most commonly gastrointestinal discomfort. In Calabrese’s study (Calabrese et al., 2008) using a general “stomach discomfort” description, there was no difference between the test substance and the placebo (*p* = 1.0). However, in Morgan’s study (Morgan and Stevens, 2010), after using a more detailed quantification method, the increase in stool frequency, nausea, and abdominal cramps were significantly higher than those of the placebo (*p* < 0.01), suggesting that detailed indicators can detect mild gastrointestinal irritation. Curcumin, caffeine-guarana, and ginkgo leaf preparations only showed mild gastrointestinal discomfort or occasional cases. The original counts were all single digits, and the differences between groups were not significant. No significant toxicity was found. Overall, no serious or dose-limiting toxicity was observed. However, due to the small sample size and short follow-up period, the long-term safety and rare interactions still require large-scale studies for verification.

**TABLE 8:** Adverse reactions and safety indicators.

Study ID	Interventions	Adverse events	p-value	Safety indicator
J. C. Galduróz	Caffeine/Guarana	Burning in the stomach (T (guarana):3, T (caffeine):1, C:0)insomnia (T:0, C:1)	NR	NR
Joseph A	Ginkgo biloba	headaches (T:5, C:4); insomnia (T:1, C:1); Gastrointestinal (T:3, C:5) Respiratory/allergic (T:2, C:3); Genitourinary (T:1, C:2); Cardiovascular (T:0,C:2) Dermatologic (T:0, C:1); Miscellaneous (T:0, C:2)	NR	NR
J. J. Carlson	Ginkgo biloba + Gotu kola + DHA	Mild adverse reactions (T:8, C:15)	NR	NR
C. Calabrese	Bacopa monnieri	Stomach upset (T:9, C:10)	p = 1.0	NR
A. Morgan	Bacopa monnieri	Gastrointestinal tract (T:35, C:8); headache (T:1, C:1); Hypertension (T:0, C:1) Insomnia (T:1, C:1)	p < 0.01	Gastrointestinal tract side-effects
G. W. Small	Curcumin	gastrointestinal (T:4, C:1)	NR	NR

Abbreviation: T, treatment group; C, control group; NR, not reported.

### 3.6 Publication bias test

Funnel plots were constructed for each outcome measure to assess potential publication bias. Visual inspection of the funnel plots revealed no significant publication bias. Further details are provided in Figure 8. Furthermore, the Gelman-Rubin convergence diagnostics (Supplementary Figures S1-S5; Supplementary Table S10) and the results of the influence analysis (Supplementary Figures S6-S10) are provided in the Supplementary Material.

**FIGURE 8:**
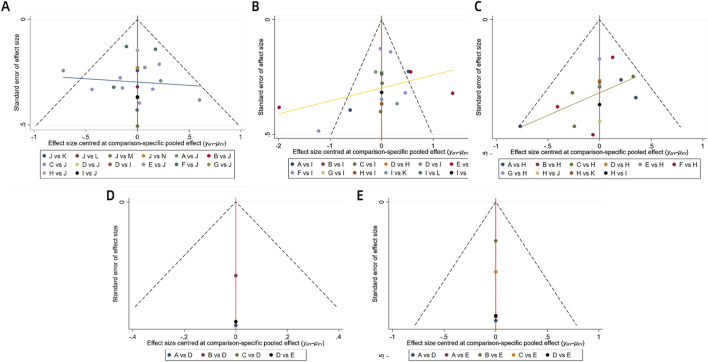
Funnel plot on publication bias. Note: **(A)** Learning and Memory; **(B)** Complex Attention; **(C)** Executive Function; **(D)** Language; **(E)** Perceptual-motor function.

## 4 Discussion

In this study, we compared the effects of various plant active substances on cognitive function improvement. A total of 25 studies were included, involving 10 plant active substances and related compound formulations, with a sample size of 1,861 healthy older adults. Our study revealed notable variations in the probability rankings of different plant active substances across cognitive domains. In the domains of learning and memory, raisin extract, tart cherry, and the Bacopa monnieri compound ranked highest. For executive function, the Bacopa monnieri compound, curcumin, and tart cherry showed relatively prominent signals of improvement. In the language domain, the Bacopa monnieri compound and raisin obtained higher ranking probabilities. Regarding perceptual-motor function, guarana ranked first. However, the two domains (language and perceptual-motor) only include 4 and 3 studies respectively, with weak evidence, and need to be interpreted with caution. Furthermore, the overall improvement in complex attention function was generally not significant. In summary, this study did not find a universally applicable best intervention for all cognitive domains; the improvement effects of different plant active substances on cognitive functions have significant domain specificity.

The results of this study indicated that raisin and tart cherry ranked highest in the probability ranking for learning and memory function. A previous meta-analysis showed that grapes could provide benefits in memory performance for both healthy individuals and those with mild cognitive impairment, which aligns with our results (Bird et al., 2022). The improvement in memory function by raisins and tart cherries may be closely linked to their high content of polyphenolic metabolites, such as anthocyanins and flavonoids (Williamson and Carughi, 2010). Studies have shown that polyphenols can regulate cognitive levels through their antioxidant, anti-inflammatory, and neuroprotective properties, via pathways such as enhancing synaptic plasticity and regulating the gut-brain axis (Arias-Sánchez et al., 2023; Rojas-García et al., 2023). Notably, although both the blueberry group and the grape-blueberry combination in this study were rich in polyphenols, their effects were weaker than those of raisins. This may be attributed to differences in intervention forms, dosages, and bioavailability. The multiple components of whole foods (such as fibers and organic acids) might work synergistically with polyphenols to enhance their beneficial effects, whereas purified extracts may show weaker outcomes (Fraga et al., 2019). This is consistent with the findings observed in a study comparing pomegranate juice and pomegranate polyphenol supplements (Kerimi et al., 2017). Furthermore, other research suggests that polyphenols require moderate doses (500 mg) and moderate bioavailability (9%–43%) to cross the blood-brain barrier and significantly impact cognitive health (Ammar et al., 2020).

The results of this study indicate that the Bacopa monnieri compound (Bacopa monnieri, Lycopene, Astaxanthin, and Vitamin B12) demonstrated relatively high potential as the optimal intervention across multiple cognitive domains, including attention, executive function, and language. This extensive improvement effect may be attributed to the multi-target synergy mechanism of its components. Bacopa monnieri has a long history of use in Ayurvedic medicine in India as a brain tonic and memory enhancer (Murthy, 2022). Its main bioactive metabolites are triterpenoid saponins, such as bacoside A and bacopaside I (Sekhar et al., 2019), which have demonstrated antioxidant and anti-inflammatory properties in preclinical studies (Dwivedi et al., 2013; Rai et al., 2015; Sumathi et al., 2012; Valotto et al., 2024). Furthermore, the other constituents in the Bacopa monnieri compound also possess their own respective benefits. Lycopene is a fat-soluble carotenoid that exerts protective effects against various central nervous system disorders and can upregulate the expression of brain-derived neurotrophic factor (BDNF) (Chen et al., 2019; Xu et al., 2025). Clinical trials on astaxanthin have indicated that it can enhance working memory and processing speed in both healthy individuals and patients with Alzheimer’s disease, while also reducing Alzheimer’s related biomarkers such as phospholipid hydroperoxides (PLOOH) (Satoh et al., 2009). Additionally, the combination of astaxanthin and lycopene can exert a synergistic antioxidant effect (Liang et al., 2009; Shi et al., 2007). Vitamin B12 is crucial for the health of the nervous system, and its deficiency is associated with various cognitive impairments (Jatoi et al., 2020). Meanwhile, we observed that in the domains of attention and executive function, the Bacopa monnieri compound exhibited better efficacy than the Bacopa monnieri extract used alone, suggesting that the combined application of different plant active substances may produce favorable effects on cognitive improvement. This is consistent with the findings of a study that revealed the neuroprotective synergistic effect of this compound preparation in vitro (Castelli et al., 2020).

The results of this study show that, besides the Bacopa monnieri compound, curcumin and tart cherry also demonstrated significant improvements in executive function. Curcumin, the main active metabolite of turmeric, is known for its prominent anti-inflammatory and antioxidant properties (Gagliardi et al., 2020), and can improve cognitive function in various models of neurological diseases (Sarker and Franks, 2018). Furthermore, a study involving middle-aged rhesus monkeys confirmed the positive effects of curcumin on executive function, with long-term supplementation enhancing spatial working memory in the monkeys (Zhang et al., 2015). Some studies have also shown that curcumin improves attention, working memory, and mood in older adults populations, delaying cognitive decline, which is consistent with our findings (Cox et al., 2015).

In terms of perceptual-motor function, our study shows that guarana ranks the highest, followed by ginkgo biloba extract. Guarana, also known as Brazilian cocoa, is rich in active compounds such as caffeine, polyphenols, and xanthines (Malik and Tlustos, 2023). In exercise-related research, guarana mouthwash was found to improve cognitive performance during submaximal exercise (Pomportes et al., 2017), which provides some evidence for its potential perceptual-motor benefits. It is important to note that the high caffeine content in guarana may cause side effects, particularly when used in excess (daily doses over 600 mg) (Turnbull et al., 2017). Ginkgo biloba is one of the most commonly used traditional medicines, and its standardized extract (EGB 761) is rich in bioactive components such as flavonoids and terpenoid lactones (Kandiah et al., 2019). Several meta-analyses have confirmed that ginkgo leaf extract demonstrates potential in improving cognition and daily living abilities in patients with Alzheimer’s disease, vascular dementia, cognitive impairment, and ischemic stroke (Chong et al., 2020; Tan et al., 2015; Thancharoen et al., 2019; Wang et al., 2020). However, it must be emphasized that the evidence for the perceptual-motor domain is the most limited in this study, with only three trials included. Consequently, the apparent advantage of guarana and Ginkgo biloba in this cognitive domain should be considered preliminary and requires urgent verification in future high-quality studies.

Finally, when interpreting the ranking results of this study comprehensively, it is essential to consider that pharmacokinetics and bioavailability are an important factor contributing to between-study heterogeneity and affecting the stability of the rankings. When exploring the potential effects of different plant active substances, their clinical outcomes often show differences due to various variables such as dosage, composition, study design, and patient characteristics (Conti et al., 2024). For example, although curcumin in this study consistently employed advanced formulations (liposomal or nanoparticle-based), differences in dosage (80 mg/d versus 180 mg/d) may lead to different effective concentrations in the body (Small et al., 2018; Cox et al., 2020). The absorption of lipid-soluble constituents in the Bacopa monnieri compound (such as lycopene and astaxanthin)depends on co-administration with dietary fats. Furthermore, the form of intervention, such as whole-food versus concentrated extract, introduces complex matrix effects that significantly influence the release and absorption of active components. Therefore, future clinical studies should more systematically report information on formulation type, timing of administration, and other relevant details, which is crucial for accurately evaluating the true effects of interventions and determining optimal dosing regimens.

## 5 Clinical value and safety considerations

Our study demonstrates that various plant active substances can improve cognitive function in healthy older adults, particularly in preventing and delaying age-related cognitive decline. Due to their multi-component synergistic effects and relative safety, natural products hold promise as a significant supplement to existing intervention strategies. Early supplementation with specific plant active substances may be an important approach to delay cognitive decline. However, their practical application requires consideration of multiple factors. Firstly, standardized tools should be used to accurately assess an individual’s cognitive state in order to match them with appropriate natural products (e.g., choosing raisin for memory decline, or a Bacopa monnieri compound for executive function deficits). Secondly, personalized plans should be developed, considering the bioavailability of active ingredients, dosage regimens, and individual health differences. Additionally, an efficacy and side-effect monitoring system should be established, complemented by lifestyle interventions such as dietary adjustments, cognitive training, and psychological support to maximize the effects of the intervention. It is important to emphasize that, while this study provides evidence for the cognitive-enhancing effects of plant active substances, their clinical translation must be approached cautiously. Current evidence largely comes from short-term, medium-sized randomized controlled trials, and data on certain cognitive domains (such as language and perceptual-motor functions) remain limited. Long-term safety and the interactions between different compounds need further exploration. Therefore, it is recommended that the use of plant active substances in clinical practice be guided by healthcare professionals, ensuring safety and individual suitability.

Furthermore, potential safety risks, especially those related to interactions with conventional medications, are a core issue that cannot be ignored in the clinical translation of this approach (Nicholson et al., 2024). The older adults involved in this study often requires long-term use of prescription drugs due to multiple comorbidities, making it crucial to assess the interactions between botanical supplements and medications. This is particularly true for older patients undergoing long-term treatment for central nervous system disorders (such as depression, Alzheimer’s disease), where such risks are more pronounced (Zabłocka-Słowińska et al., 2013). For example, in patients with Alzheimer’s disease, B-vitamin supplements have a certain risk of triggering depressive disorders and delirium (Conti et al., 2025). Although the randomized controlled trials included in this study reported good safety and rare serious adverse events, these short-term trials typically set strict exclusion criteria and may not fully reflect the complex situations in the real world with long-term, multi-drug combination use. Current pharmacological studies have indicated certain potential interaction risks. For instance, ginkgo biloba extract, due to its anti-platelet aggregation activity, may theoretically increase the risk of bleeding when used in combination with anticoagulants such as warfarin and aspirin. However, the risk is closely related to the standardization of the extract, and studies have shown that a daily dose of ginkgo biloba extract (such as EGb 761 standard) not exceeding 240 mg has a low risk of clinically significant drug interactions (Unger, 2013). Therefore, based on current evidence, we strongly recommend: older adults, especially those with chronic diseases undergoing medication treatment, should consult a doctor or clinical pharmacist before starting to take any plant active substance supplements for long-term use, and undergo a comprehensive benefit-risk assessment. Future research urgently needs to conduct pharmacoepidemiological studies and design rigorous clinical trials on drug interactions to further clarify the long-term safety of these natural products in real medical settings.

## 6 Strengths and limitations

Our study has several strengths. Firstly, it encompasses 25 randomized controlled trials involving 1,861 healthy older adults, providing a significant sample size. Secondly, we compared 10 different natural active substances, applying strict inclusion criteria, systematically searching for all relevant studies that met predefined conditions, and reporting in accordance with systematic review and meta-analysis guidelines. However, our study also has certain limitations. Firstly, there were fewer studies on certain cognitive domains, such as language and perceptual-motor functions (only 4 and 3 studies, respectively), limiting the strength of the evidence. Secondly, there is heterogeneity in the dosage, duration of intervention, and assessment tools used, which may affect the consistency of the results. Thirdly, variations in the extraction methods of different plant active substances may introduce some bias into our findings. Therefore, we recommend that readers interpret our conclusions with caution.

## 7 Conclusion

The findings of this study indicate that raisin and tart cherry show relatively favorable interventional potential in the domain of memory function, whereas the Bacopa monnieri compound preparation demonstrates greater advantage for executive function. However, the active constituents and mechanisms of action of these three interventions differ markedly, and their effects should be interpreted within specific cognitive domains rather than directly compared or generalized. At present, most available studies are short-term interventions with moderate sample sizes; moreover, evidence in the language and perceptual-motor domains is extremely limited, and considerable heterogeneity exists among cognitive assessment tools. Therefore, the current strength of evidence should be viewed with caution. Therefore, future research should consider: (1) conducting high-quality, long-term follow-up randomized controlled trials to further validate the safety and efficacy of these interventions; (2) optimizing extraction methods and establishing the ideal dosage based on the bioavailability of different plant active substances; and (3) exploring the molecular mechanisms of synergistic effects in the combined use of different plant active substances.

## Data Availability

The original contributions presented in the study are included in the article/Supplementary Material, further inquiries can be directed to the corresponding author.

## References

[B1] Ammar A. Trabelsi K. Müller P. Bouaziz B. Boukhris O. Glenn J. M. (2020). The effect of (Poly)phenol-Rich interventions on cognitive functions and neuroprotective measures in healthy aging adults: a systematic review and meta-analysis. J. Clin. Med. 9 (3), 835. 10.3390/jcm9030835 32204500 PMC7141326

[B2] Arias-Sánchez R. A. Torner L. Fenton N. B. (2023). Polyphenols and neurodegenerative diseases: potential effects and mechanisms of neuroprotection. Molecules 28 (14), 5415. 10.3390/molecules28145415 37513286 PMC10385962

[B3] Baranowska-Wojcik E. Gajowniczek-Alasa D. Pawlikowska-Pawlega B. Szwajgier D. (2025). The potential role of phytochemicals in alzheimer's disease. Nutrients 17 (4), 653. 10.3390/nu17040653 40004981 PMC11858096

[B4] Bensalem J. Dudonné S. Etchamendy N. Pellay H. Amadieu C. Gaudout D. (2019). Polyphenols from grape and blueberry improve episodic memory in healthy elderly with lower level of memory performance: a bicentric double-blind, randomized, placebo-controlled clinical study. Journals Gerontology Ser. A 74 (7), 996–1007. 10.1093/gerona/gly166 30032176

[B5] Bird R. J. Hoggard N. Aceves-Martins M. (2022). The effect of grape interventions on cognitive and mental performance in healthy participants and those with mild cognitive impairment: a systematic review of randomized controlled trials. Nutr. Rev. 80 (3), 367–380. 10.1093/nutrit/nuab025 34041549 PMC8829676

[B6] Bowtell J. L. Aboo-Bakkar Z. Conway M. E. Adlam A. R. Fulford J. (2017). Enhanced task-related brain activation and resting perfusion in healthy older adults after chronic blueberry supplementation. Appl. Physiology, Nutr. Metabolism 42 (7), 773–779. 10.1139/apnm-2016-0550 28249119

[B7] Calabrese C. Gregory W. L. Leo M. Kraemer D. Bone K. Oken B. (2008). Effects of a StandardizedBacopa monnieri extract on cognitive performance, anxiety, and depression in the elderly: a randomized, double-blind, placebo-controlled trial. J. Altern. Complementary Med. 14 (6), 707–713. 10.1089/acm.2008.0018 18611150 PMC3153866

[B8] Carlson J. J. Farquhar J. W. Dinucci E. Ausserer L. Zehnder J. Miller D. (2007). Safety and efficacy of a ginkgo biloba–containing dietary supplement on cognitive function, quality of life, and platelet function in healthy, cognitively intact older adults. J. Am. Dietetic Assoc. 107 (3), 422–432. 10.1016/j.jada.2006.12.011 17324660

[B9] Castelli V. Melani F. Ferri C. D'Angelo M. Catanesi M. Grassi D. (2020). Neuroprotective activities of bacopa, lycopene, astaxanthin, and vitamin B12 combination on oxidative stress-dependent neuronal death. J. Cell. Biochem. 121 (12), 4862–4869. 10.1002/jcb.29722 32449987

[B10] Chai S. C. Jerusik J. Davis K. Wright R. S. Zhang Z. (2019). Effect of montmorency tart cherry juice on cognitive performance in older adults: a randomized controlled trial. Food Funct. 1 (7), 4423–4431. 10.1039/c9fo00913b 31287117

[B11] Chaimani A. Higgins J. Mavridis D. Spyridonos P. Salanti G. (2013). Graphical tools for network meta-analysis in STATA. PLoS One 8 (10), 1–12. 10.1371/journal.pone.0076654 24098547 PMC3789683

[B12] Chen D. Huang C. Chen Z. (2019). A review for the pharmacological effect of lycopene in central nervous system disorders. Biomed. Pharmacother. 111, 791–801. 10.1016/j.biopha.2018.12.151 30616078

[B13] Cheng N. Barfoot K. L. Le Cozannet R. Fança-Berthon P. Lamport D. J. Williams C. M. (2024). Wild blueberry extract intervention in healthy older adults: a multi-study, randomised, controlled investigation of acute cognitive and cardiovascular effects. Nutrients 16 (8), 1180. 10.3390/nu16081180 38674870 PMC11054866

[B14] Chong P. Z. Ng H. Y. Tai J. T. Lee S. (2020). Efficacy and safety of Ginkgo biloba in patients with acute ischemic stroke: a systematic review and meta-analysis. Am. J. Chin. Med. 48 (3), 513–534. 10.1142/S0192415X20500263 32349519

[B15] Conti V. Polcaro G. De Bellis E. Donnarumma D. De Rosa F. Stefanelli B. (2024). Natural health products for anti-cancer treatment: evidence and controversy. J. Pers. Med. 14 (7), 685. 10.3390/jpm14070685 39063939 PMC11278393

[B16] Conti V. Zarrella A. Donnarumma D. Pagano A. Mazza I. De Stefano A. (2025). Natural health products in the prevention and management of alzheimer’s disease: a systematic review of randomized clinical trials. Appl. Sci. 15 (7), 3513. 10.3390/app15073513

[B17] Cox K. H. Pipingas A. Scholey A. B. Nutt D. J. Blier P. (2015). Investigation of the effects of solid lipid curcumin on cognition and mood in a healthy older population. J. Psychopharmacology Oxf. 29 (5), 642–651. 10.1177/0269881114552744 25277322

[B18] Cox K. H. M. White D. J. Pipingas A. Poorun K. Scholey A. (2020). Further evidence of benefits to mood and working memory from lipidated curcumin in healthy older people: a 12-Week, double-blind, placebo-controlled, partial replication study. Nutrients 12 (6), 1678. 10.3390/nu12061678 32512782 PMC7352411

[B19] Cropley V. Croft R. Silber B. Neale C. Scholey A. Stough C. (2012). Does coffee enriched with chlorogenic acids improve mood and cognition after acute administration in healthy elderly? A pilot study. Psychopharmacologia. 219 (3), 737–749. 10.1007/s00213-011-2395-0 21773723

[B20] Crosta F. Stefani A. Melani F. Fabrizzi P. Nizzardo A. Grassi D. (2021). Improvement of executive function after short-term administration of an antioxidants mix containing bacopa, lycopene, astaxanthin and vitamin B12: the BLAtwelve study. Nutrients 13 (1), 56. 10.3390/nu13010056 33375429 PMC7824614

[B21] Dwivedi S. Nagarajan R. Hanif K. Siddiqui H. H. Nath C. Shukla R. (2013). Standardized extract of bacopa monniera attenuates okadaic acid induced memory dysfunction in rats: effect on Nrf2 pathway. Evid.-based Complement. Altern. Med. 2013, 294501. 10.1155/2013/294501 24078822 PMC3776558

[B22] Fraga C. G. Croft K. D. Kennedy D. O. Tomás-Barberán F. A. (2019). The effects of polyphenols and other bioactives on human health. Food Funct. 10 (2), 514–528. 10.1039/c8fo01997e 30746536

[B23] Gagliardi S. Morasso C. Stivaktakis P. Pandini C. Tinelli V. Tsatsakis A. (2020). Curcumin formulations and trials: what's new in neurological diseases. Molecules 25 (22), 5389. 10.3390/molecules25225389 33217959 PMC7698610

[B24] Galduroz J. C. Carlini E. A. (1996). The effects of long-term administration of guarana on the cognition of normal, elderly volunteers. Sao Paulo Med. J. 114 (1), 1073–1078. 10.1590/s1516-31801996000100003 8984582

[B25] Gausemel Å. Filkuková P. (2025). Innovations in dementia screening: a systematic review and meta-analysis of virtual reality assessments. Front. Psychol. 16, 1606562. 10.3389/fpsyg.2025.1606562 41293102 PMC12640857

[B26] Hack B. Penna E. M. Talik T. Chandrashekhar R. Millard-Stafford M. (2023). Effect of guarana (Paullinia cupana) on cognitive performance: a systematic review and meta-analysis. Nutrients 15 (2), 434. 10.3390/nu15020434 36678305 PMC9865053

[B27] Heinrich M. Jalil B. (2023). From the CONSORT to the ConPhyMP statement and beyond-how to ascertain best practice. Front. Pharmacol. 14, 1338710. 10.3389/fphar.2023.1338710 38149050 PMC10750347

[B28] Heinrich M. Jalil B. Abdel-Tawab M. Echeverria J. Kulić Ž. Mcgaw L. J. (2022). Best practice in the chemical characterisation of extracts used in pharmacological and toxicological research-The ConPhyMP-Guidelines. Front. Pharmacol. 13, 953205. 10.3389/fphar.2022.953205 36176427 PMC9514875

[B29] Jackson D. Riley R. White I. R. (2011). Multivariate meta-analysis: potential and promise. Stat. Med. 30 (20), 2481–2498. 10.1002/sim.4172 21268052 PMC3470931

[B30] Jatoi S. Hafeez A. Riaz S. U. Ali A. Ghauri M. I. Zehra M. (2020). Low vitamin B12 levels: an underestimated cause of minimal cognitive impairment and dementia. Cureus 12 (2), e6976. 10.7759/cureus.6976 32206454 PMC7077099

[B31] Kandiah N. Ong P. A. Yuda T. Ng L. L. Mamun K. Merchant R. A. (2019). Treatment of dementia and mild cognitive impairment with or without cerebrovascular disease: expert consensus on the use of Ginkgo biloba extract, EGb 761(®). CNS Neurosci. Ther. 25 (2), 288–298. 10.1111/cns.13095 30648358 PMC6488894

[B32] Kerimi A. Nyambe-Silavwe H. Gauer J. S. Tomás-Barberán F. A. Williamson G. (2017). Pomegranate juice, but not an extract, confers a lower glycemic response on a high-glycemic index food: randomized, crossover, controlled trials in healthy subjects. Am. J. Clin. Nutr. 106 (6), 1384–1393. 10.3945/ajcn.117.161968 29021286

[B33] Liang J. Tian Y. Yang F. Zhang J. Skibsted L. H. (2009). Antioxidant synergism between carotenoids in membranes. Astaxanthin as a radical transfer bridge. Food Chem. 115 (4), 1437–1442. 10.1016/j.foodchem.2009.01.074

[B34] Lorca C. Mulet M. Arevalo-Caro C. Sanchez M. A. Perez A. Perrino M. (2023). Plant-derived nootropics and human cognition: a systematic review. Crit. Rev. Food. Sci. Nutr. 63 (22), 5521–5545. 10.1080/10408398.2021.2021137 34978226

[B35] Lunny C. Higgins J. White I. R. Dias S. Hutton B. Wright J. M. (2025). Risk of bias in network meta-analysis (RoB NMA) tool. BMJ 388, e079839. 10.1136/bmj-2024-079839 40101916 PMC11915405

[B36] Malik M. Tlustos P. (2023). Nootropic herbs, shrubs, and trees as potential cognitive enhancers. Plants 12 (6), 1364. 10.3390/plants12061364 36987052 PMC10056569

[B37] Marotta N. Demeco A. Moggio L. Marinaro C. Pino I. Barletta M. (2020). Comparative effectiveness of breathing exercises in patients with chronic obstructive pulmonary disease. Complement. Ther. Clin. Pract. 41, 101260. 10.1016/j.ctcp.2020.101260 33221632

[B38] Mcnamara R. K. Kalt W. Shidler M. D. Mcdonald J. Summer S. S. Stein A. L. (2018). Cognitive response to fish oil, blueberry, and combined supplementation in older adults with subjective cognitive impairment. Neurobiol. Aging 64, 147–156. 10.1016/j.neurobiolaging.2017.12.003 29458842 PMC5822748

[B39] Mcphee G. M. Downey L. A. Wesnes K. A. Stough C. (2021). The neurocognitive effects of Bacopa monnieri and cognitive training on markers of brain microstructure in healthy older adults. Front. Aging Neurosci. 13, 638109. 10.3389/fnagi.2021.638109 33692683 PMC7937913

[B40] Miller M. G. Hamilton D. A. Joseph J. A. Shukitt-Hale B. (2018). Dietary blueberry improves cognition among older adults in a randomized, double-blind, placebo-controlled trial. Eur. J. Nutr. 57 (3), 1169–1180. 10.1007/s00394-017-1400-8 28283823

[B41] Mix J. A. Crews W. J. (2000). An examination of the efficacy of Ginkgo biloba extract EGb761 on the neuropsychologic functioning of cognitively intact older adults. J. Altern. Complement. Med. 6 (3), 219–229. 10.1089/acm.2000.6.219 10890330

[B42] Mix J. A. David Crews W. (2002). A double‐blind, placebo‐controlled, randomized trial ofGinkgo biloba extract EGb 761® in a sample of cognitively intact older adults: neuropsychological findings. Hum. Psychopharmacol. Clin. Exp. 17 (6), 267–277. 10.1002/hup.412 12404671

[B43] Moher D. Shamseer L. Clarke M. Ghersi D. Liberati A. Petticrew M. (2015). Preferred reporting items for systematic review and meta-analysis protocols (PRISMA-P) 2015 statement. Syst. Rev. 4 (1), 1. 10.1186/2046-4053-4-1 25554246 PMC4320440

[B44] Morgan A. Stevens J. (2010). DoesBacopa monnieri improve memory performance in older persons? Results of a randomized, placebo-controlled, double-blind trial. J. Altern. Complementary Med. 16 (7), 753–759. 10.1089/acm.2009.0342 20590480

[B45] Murthy H. N. (2022). Biotechnological production of bacosides from cell and organ cultures of Bacopa monnieri. Appl. Microbiol. Biotechnol. 106 (5-6), 1799–1811. 10.1007/s00253-022-11834-0 35201388

[B46] Nathan P. J. Ricketts E. Wesnes K. Mrazek L. Greville W. Stough C. (2002). The acute nootropic effects ofGinkgo bilobain healthy older human subjects: a preliminary investigation. Hum. Psychopharmacol. Clin. Exp. 17 (1), 45–49. 10.1002/hup.353 12404706

[B47] Nicholson K. Liu W. Fitzpatrick D. Hardacre K. A. Roberts S. Salerno J. (2024). Prevalence of multimorbidity and polypharmacy among adults and older adults: a systematic review. Lancet. Healthy Longevity 5 (4), e287–e296. 10.1016/S2666-7568(24)00007-2 38452787

[B48] Nikolakopoulou A. Higgins J. Papakonstantinou T. Chaimani A. Del G. C. Egger M. (2020). CINeMA: an approach for assessing confidence in the results of a network meta-analysis. PLoS Med. 17 (4), e1003082. 10.1371/journal.pmed.1003082 32243458 PMC7122720

[B49] Olejnik P. Golenia A. Malyszko J. (2025). The potential role of microbiota in age-related cognitive decline: a narrative review of the underlying molecular mechanisms. Int. J. Mol. Sci. 26 (4), 1590. 10.3390/ijms26041590 40004055 PMC11855389

[B50] Pagotto G. Santos L. Osman N. Lamas C. B. Laurindo L. F. Pomini K. T. (2024). Ginkgo biloba: a leaf of hope in the fight against alzheimer's dementia: clinical trial systematic review. Antioxidants 13 (6), 651. 10.3390/antiox13060651 38929090 PMC11201198

[B51] Papakonstantinou T. Nikolakopoulou A. Higgins J. Egger M. Salanti G. (2020). CINeMA: software for semiautomated assessment of the confidence in the results of network meta-analysis. Campbell Syst. Rev. 16 (1), e1080. 10.1002/cl2.1080 37131978 PMC8356302

[B52] Perry N. S. L. Menzies R. Hodgson F. Wedgewood P. Howes M. J. R. Brooker H. J. (2018). A randomised double-blind placebo-controlled pilot trial of a combined extract of sage, rosemary and melissa, traditional herbal medicines, on the enhancement of memory in normal healthy subjects, including influence of age. Phytomedicine 39, 42–48. 10.1016/j.phymed.2017.08.015 29433682

[B53] Pomportes L. Brisswalter J. Casini L. Hays A. Davranche K. (2017). Cognitive performance enhancement induced by caffeine, carbohydrate and guarana mouth rinsing during submaximal exercise. Nutrients 9 (6), 589. 10.3390/nu9060589 28598402 PMC5490568

[B54] Rai R. Singh H. K. Prasad S. (2015). A special extract of Bacopa monnieri (CDRI-08) restores learning and memory by upregulating expression of the NMDA receptor subunit GluN2B in the brain of scopolamine-induced amnesic mice. Evid.-based Complement. Altern. Med. 2015, 254303. 10.1155/2015/254303 26413117 PMC4564605

[B55] Roberts R. Knopman D. S. (2013). Classification and epidemiology of MCI. Clin. Geriatr. Med. 29 (4), 753–772. 10.1016/j.cger.2013.07.003 24094295 PMC3821397

[B56] Rodrigo-Gonzalo M. J. González-Manzano S. Pablos-Hernández M. C. Méndez-Sánchez R. Ayuda Duran B. González-Sánchez J. (2023). Effects of a raisin supplement on cognitive performance, quality of life, and functional activities in healthy older adults. Randomized clinical trial. Nutrients 15 (12), 2811. 10.3390/nu15122811 37375715 PMC10301980

[B57] Rojas-García A. Fernández-Ochoa Á. Cádiz-Gurrea M. L. Arráez-Román D. Segura-Carretero A. (2023). Neuroprotective effects of agri-food By-Products rich in phenolic compounds. Nutrients 15 (2), 449. 10.3390/nu15020449 36678322 PMC9865516

[B58] Rudnicka E. Napierala P. Podfigurna A. Meczekalski B. Smolarczyk R. Grymowicz M. (2020). The world health organization (WHO) approach to healthy ageing. Maturitas 139, 6–11. 10.1016/j.maturitas.2020.05.018 32747042 PMC7250103

[B59] Sachdev P. S. Blacker D. Blazer D. G. Ganguli M. Jeste D. V. Paulsen J. S. (2014). Classifying neurocognitive disorders: the DSM-5 approach. Nat. Rev. Neurol. 10 (11), 634–642. 10.1038/nrneurol.2014.181 25266297

[B60] Salanti G. Ades A. E. Ioannidis J. P. (2011). Graphical methods and numerical summaries for presenting results from multiple-treatment meta-analysis: an overview and tutorial. J. Clin. Epidemiol. 64 (2), 163–171. 10.1016/j.jclinepi.2010.03.016 20688472

[B61] Sarker M. R. Franks S. F. (2018). Efficacy of curcumin for age-associated cognitive decline: a narrative review of preclinical and clinical studies. GeroScience 40 (2), 73–95. 10.1007/s11357-018-0017-z 29679204 PMC5964053

[B62] Satoh A. Tsuji S. Okada Y. Murakami N. Urami M. Nakagawa K. (2009). Preliminary clinical evaluation of toxicity and efficacy of A new astaxanthin-rich Haematococcus pluvialis extract. J. Clin. Biochem. Nutr. 44 (3), 280–284. 10.3164/jcbn.08-238 19430618 PMC2675019

[B63] Scholey A. B. Tildesley N. T. J. Ballard C. G. Wesnes K. A. Tasker A. Perry E. K. (2008). An extract of salvia (sage) with anticholinesterase properties improves memory and attention in healthy older volunteers. Psychopharmacologia 198 (1), 127–139. 10.1007/s00213-008-1101-3 18350281

[B64] Sekhar V. C. Viswanathan G. Baby S. (2019). Insights into the molecular aspects of neuroprotective bacoside A and bacopaside I. Curr. Neuropharmacol. 17 (5), 438–446. 10.2174/1570159X16666180419123022 29676230 PMC6520587

[B65] Sharma K. (2019). Cholinesterase inhibitors as Alzheimer's therapeutics. Mol. Med. Rep. 20 (2), 1479–1487. 10.3892/mmr.2019.10374 31257471 PMC6625431

[B66] Shi J. Qu Q. Kakuda Y. Xue S. J. Jiang Y. Koide S. (2007). Investigation of the antioxidant and synergistic activity of lycopene and other natural antioxidants using LAME and AMVN model systems. J. Food Compos. Anal. 20 (7), 603–608. 10.1016/j.jfca.2007.03.004

[B67] Shim S. Yoon B. H. Shin I. S. Bae J. M. (2017). Network meta-analysis: application and practice using stata. Epidemiol. Health 39, e2017047. 10.4178/epih.e2017047 29092392 PMC5733388

[B68] Small G. W. Siddarth P. Li Z. Miller K. J. Ercoli L. Emerson N. D. (2018). Memory and brain amyloid and tau effects of a bioavailable form of curcumin in non-demented adults: a double-blind, placebo-controlled 18-Month trial. Am. J. Geriatric Psychiatry 26 (3), 266–277. 10.1016/j.jagp.2017.10.010 29246725

[B69] Solomon P. R. Adams F. Silver A. Zimmer J. Deveaux R. (2002). Ginkgo for memory enhancement: a randomized controlled trial. JAMA 288 (7), 835–840. 10.1001/jama.288.7.835 12186600

[B70] Sumathi T. Shobana C. Christinal J. Anusha C. (2012). Protective effect of bacopa monniera on methyl mercury-induced oxidative stress in cerebellum of rats. Cell. Mol. Neurobiol. 32 (6), 979–987. 10.1007/s10571-012-9813-7 22366895 PMC11498485

[B71] Tan M. S. Yu J. T. Tan C. C. Wang H. F. Meng X. F. Wang C. (2015). Efficacy and adverse effects of Ginkgo biloba for cognitive impairment and dementia: a systematic review and meta-analysis. J. Alzheimers Dis. 43 (2), 589–603. 10.3233/JAD-140837 25114079

[B72] Thancharoen O. Limwattananon C. Waleekhachonloet O. Rattanachotphanit T. Limwattananon P. Limpawattana P. (2019). Ginkgo biloba extract (EGb761), cholinesterase inhibitors, and memantine for the treatment of mild-to-moderate alzheimer's disease: a network meta-analysis. Drugs. Aging. 36 (5), 435–452. 10.1007/s40266-019-00648-x 30937879

[B73] Travica N. D'Cunha N. M. Naumovski N. Kent K. Mellor D. D. Firth J. (2020). The effect of blueberry interventions on cognitive performance and mood: a systematic review of randomized controlled trials. Brain. Behav. Immun. 85, 96–105. 10.1016/j.bbi.2019.04.001 30999017

[B74] Turnbull D. Rodricks J. V. Mariano G. F. Chowdhury F. (2017). Caffeine and cardiovascular health. Regul. Toxicol. Pharmacol. 89, 165–185. 10.1016/j.yrtph.2017.07.025 28756014

[B75] Unger M. (2013). Pharmacokinetic drug interactions involving Ginkgo biloba. Drug Metab. Rev. 45 (3), 353–385. 10.3109/03602532.2013.815200 23865865

[B76] Valotto N. L. Reverete D. A. M. Moretti J. R. Mendes M. N. Joshi R. K. Dos S. B. D. (2024). Investigating the neuroprotective and cognitive-enhancing effects of bacopa monnieri: a systematic review focused on inflammation, oxidative stress, mitochondrial dysfunction, and apoptosis. Antioxidants 13 (4), 393. 10.3390/antiox13040393 38671841 PMC11047749

[B77] Vats D. Flegal J. M. Jones G. L. (2019). Multivariate output analysis for markov chain monte carlo. Biometrika 106 (2), 321–337. 10.1093/biomet/asz002

[B78] Wang M. Peng H. Peng Z. Huang K. Li T. Li L. (2020). Efficacy and safety of ginkgo preparation in patients with vascular dementia: a protocol for systematic review and meta-analysis. Med. Baltim. 99 (37), e22209. 10.1097/MD.0000000000022209 32925798 PMC7489658

[B79] Williamson G. Carughi A. (2010). Polyphenol content and health benefits of raisins. Nutr. Res. 30 (8), 511–519. 10.1016/j.nutres.2010.07.005 20851304

[B80] Wong R. H. X. Howe P. R. C. Bryan J. Coates A. M. Buckley J. D. Berry N. M. (2012). Chronic effects of a wild green oat extract supplementation on cognitive performance in older adults: a randomised, double-blind, placebo-controlled, crossover trial. Nutrients 4 (5), 331–342. 10.3390/nu4050331 22690320 PMC3367260

[B81] Wood E. Hein S. Mesnage R. Fernandes F. Abhayaratne N. Xu Y. (2023). Wild blueberry (poly)phenols can improve vascular function and cognitive performance in healthy older individuals: a double-blind randomized controlled trial. Am. J. Clin. Nutr. 117 (6), 1306–1319. 10.1016/j.ajcnut.2023.03.017 36972800 PMC10315404

[B82] Xu H. Wang Y. Geng D. Chen F. Chen Y. Niwenahisemo L. C. (2025). Lycopene alleviates depression-like behavior in chronic social defeat stress-induced mice by promoting synaptic plasticity via the BDNF-TrkB pathway. Food Sci. Nutr. 13 (1), e70003. 10.1002/fsn3.70003 39844795 PMC11751711

[B83] Zabłocka-Słowińska K. Jawna K. Biernat J. (2013). Interactions between synthetic drugs used in treatment of selected central nervous system disorders and dietary supplements and herbal drugs. Psychiatr. Pol. 47 (3), 487–498. 23885542

[B84] Zhang L. Fang Y. Xu Y. Lian Y. Xie N. Wu T. (2015). Curcumin improves amyloid beta-peptide (1-42) induced spatial memory deficits through BDNF-ERK signaling pathway. PLoS One 10 (6), e0131525. 10.1371/journal.pone.0131525 26114940 PMC4482657

