# Supplementary material for: The effect of plant active substances on cognitive function in healthy older adults: a systematic review and network meta-analysis of randomized controlled trials
Source: Front Pharmacol. 2026 Jan 20;16:1672171. doi: 10.3389/fphar.2025.1672171 (PMC12864429; doi:10.3389/fphar.2025.1672171)
Supplement: Supplementary file 2 [file Supplementaryfile1.docx]

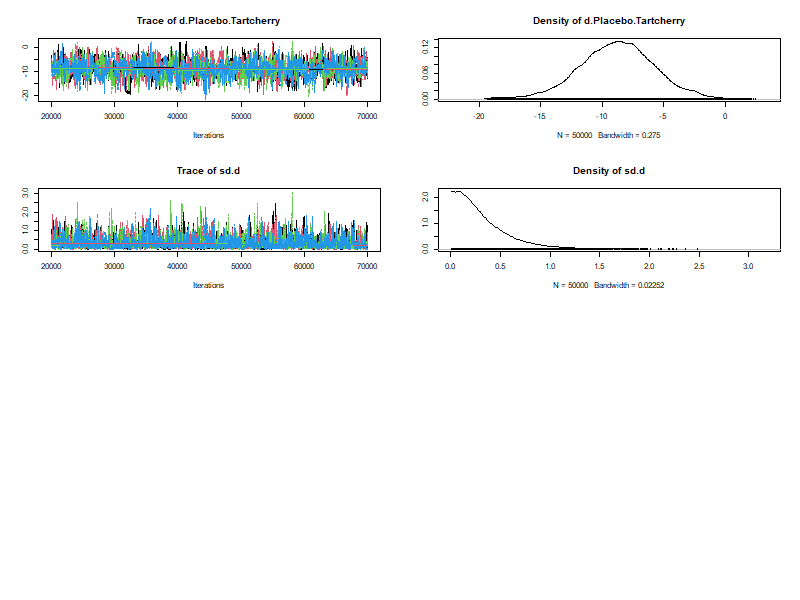
**Figure 1A Trace and density convergence diagnostics for Learning and memory.**


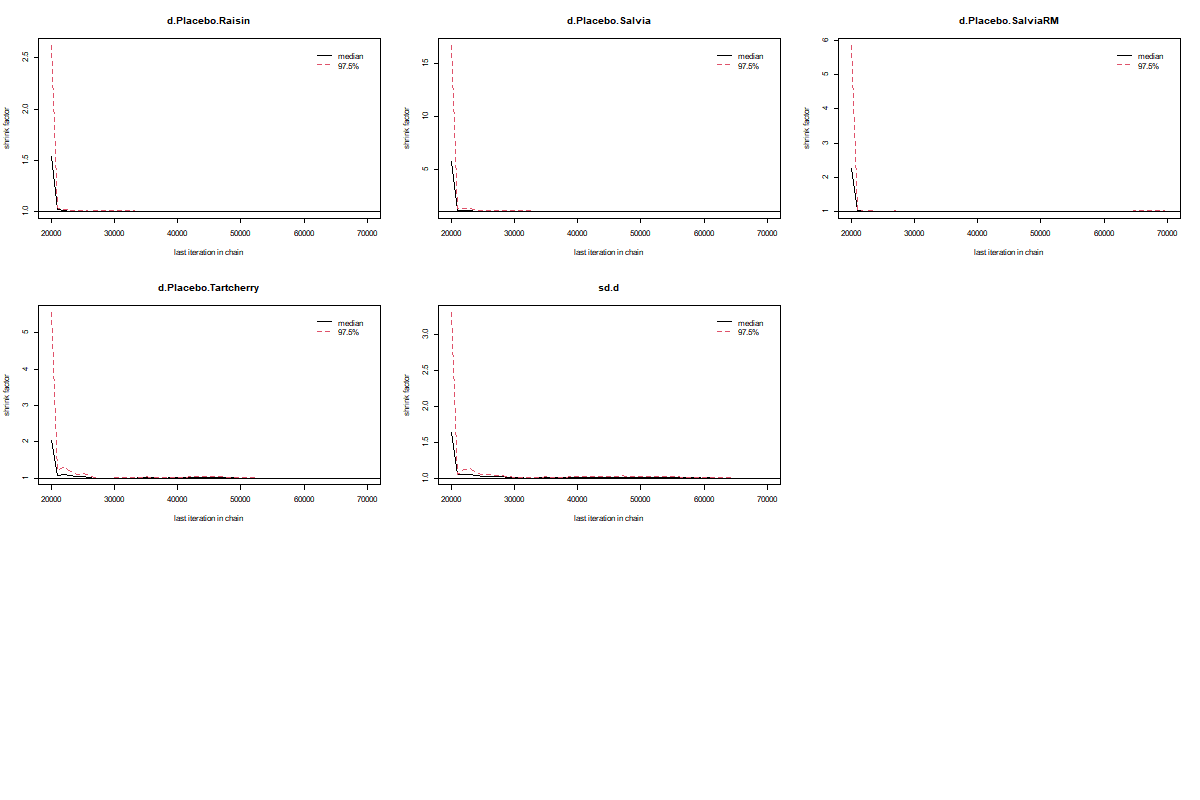


**Figure 1B Gelman-Rubin convergence diagnostics for Learning and memory.**


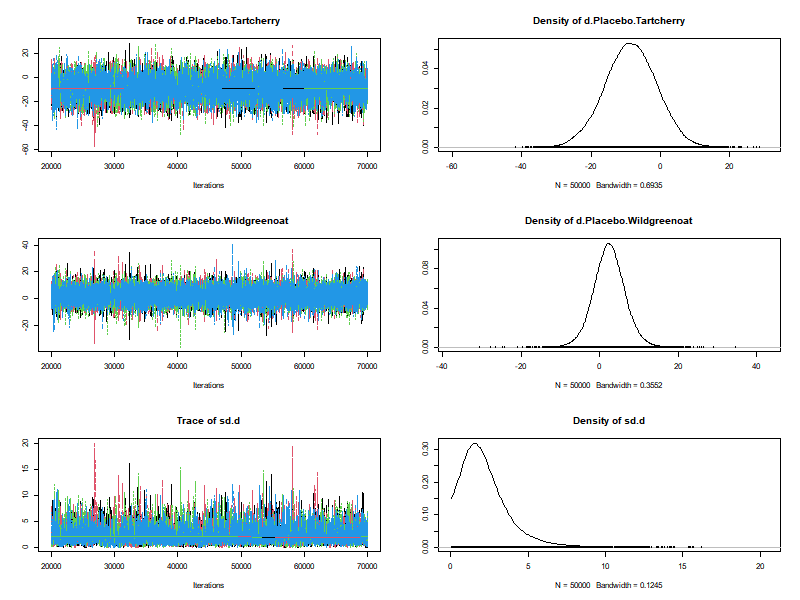


**Figure 2A Trace and density convergence diagnostics for Complex attention.**


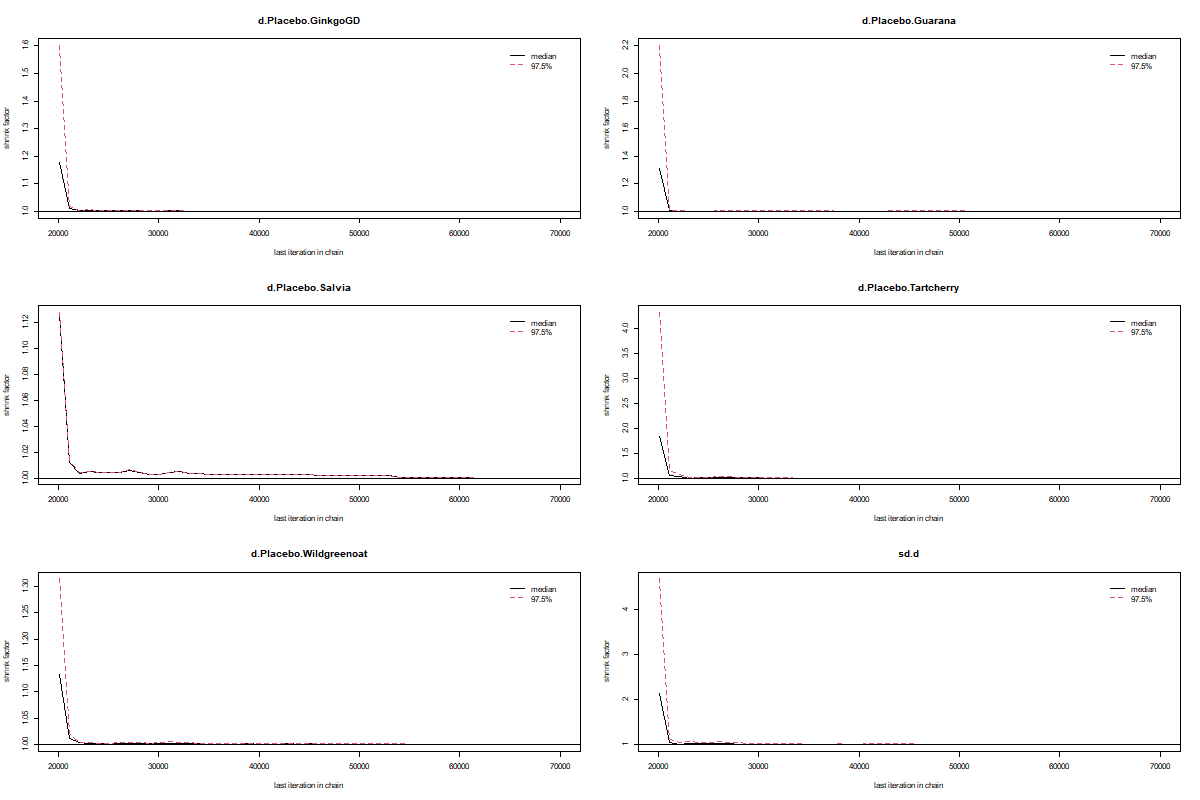


**Figure 2B Gelman-Rubin convergence diagnostics for Complex attention.**


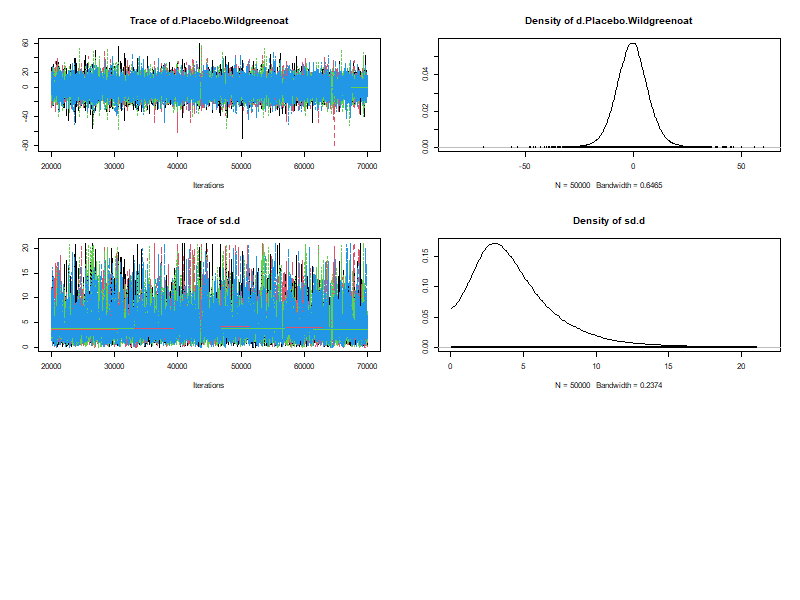


**Figure 3A Trace and density convergence diagnostics for Executive function.**


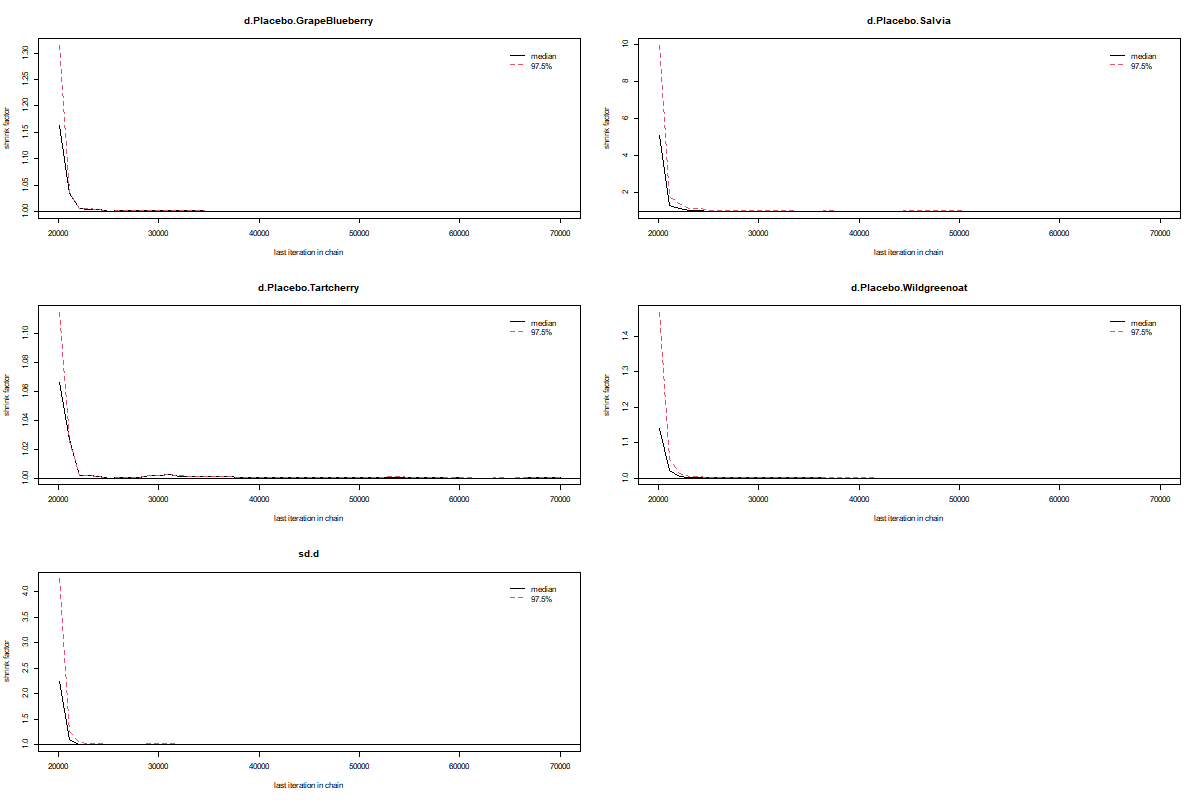


**Figure 3B Gelman-Rubin convergence diagnostics for Executive function.**


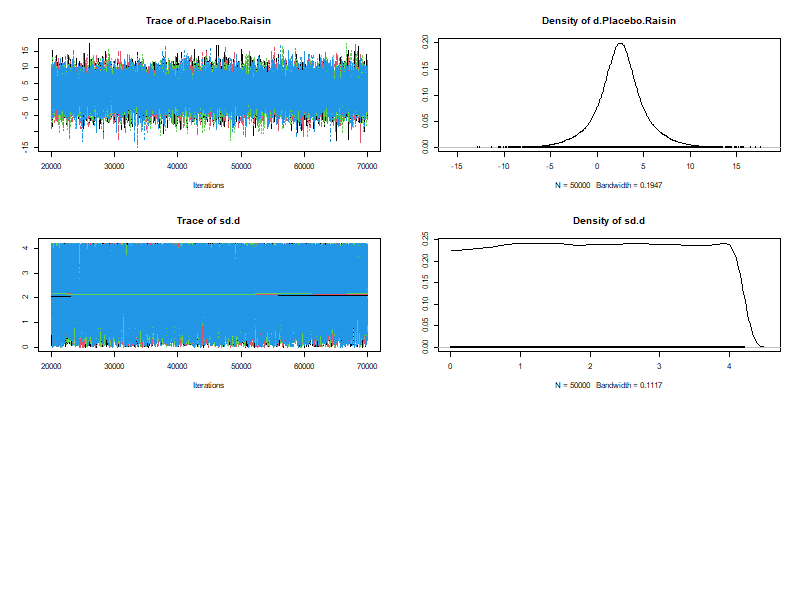


**Figure 4A Trace and density convergence diagnostics for Language.**


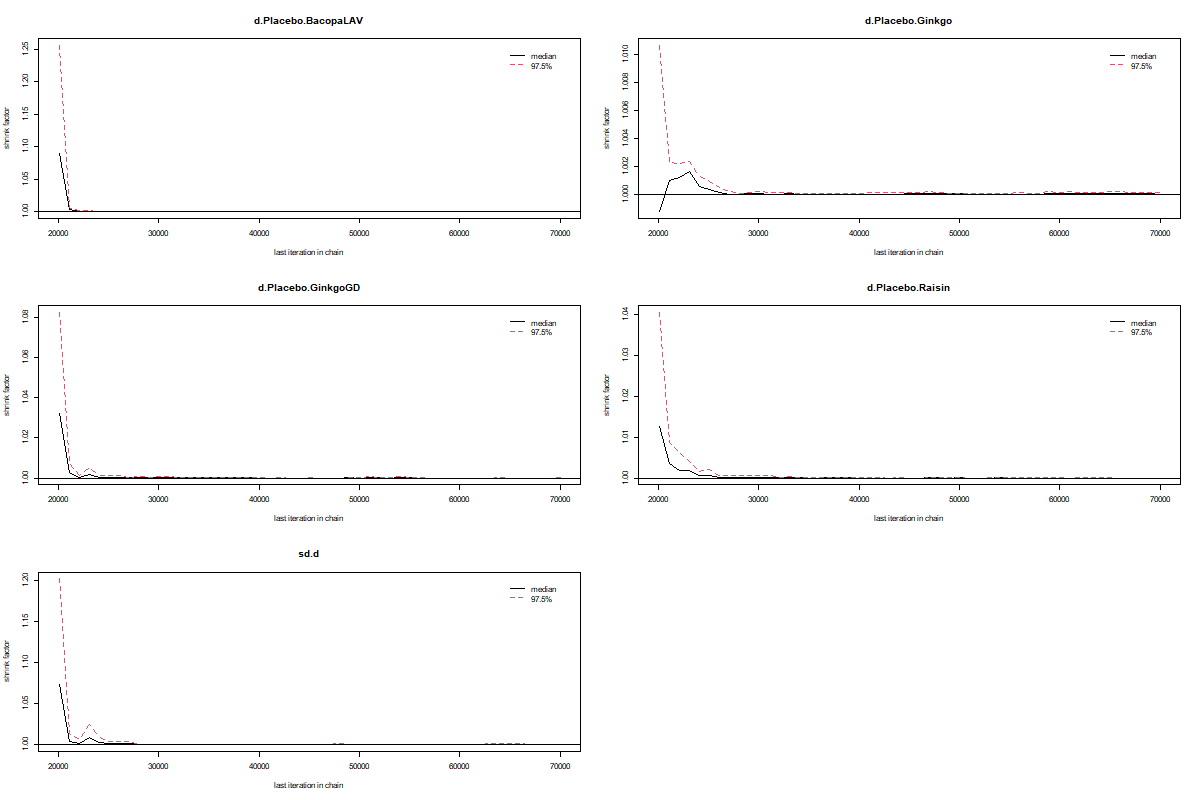


**Figure4B Gelman-Rubin convergence diagnostics for Language.**


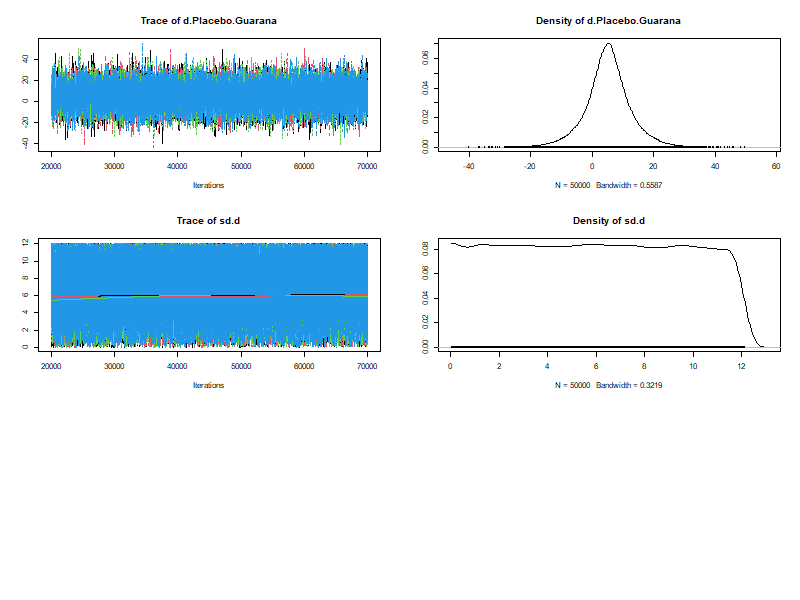


**Figure 5A Trace and density convergence diagnostics for Perceptual-motor function.**


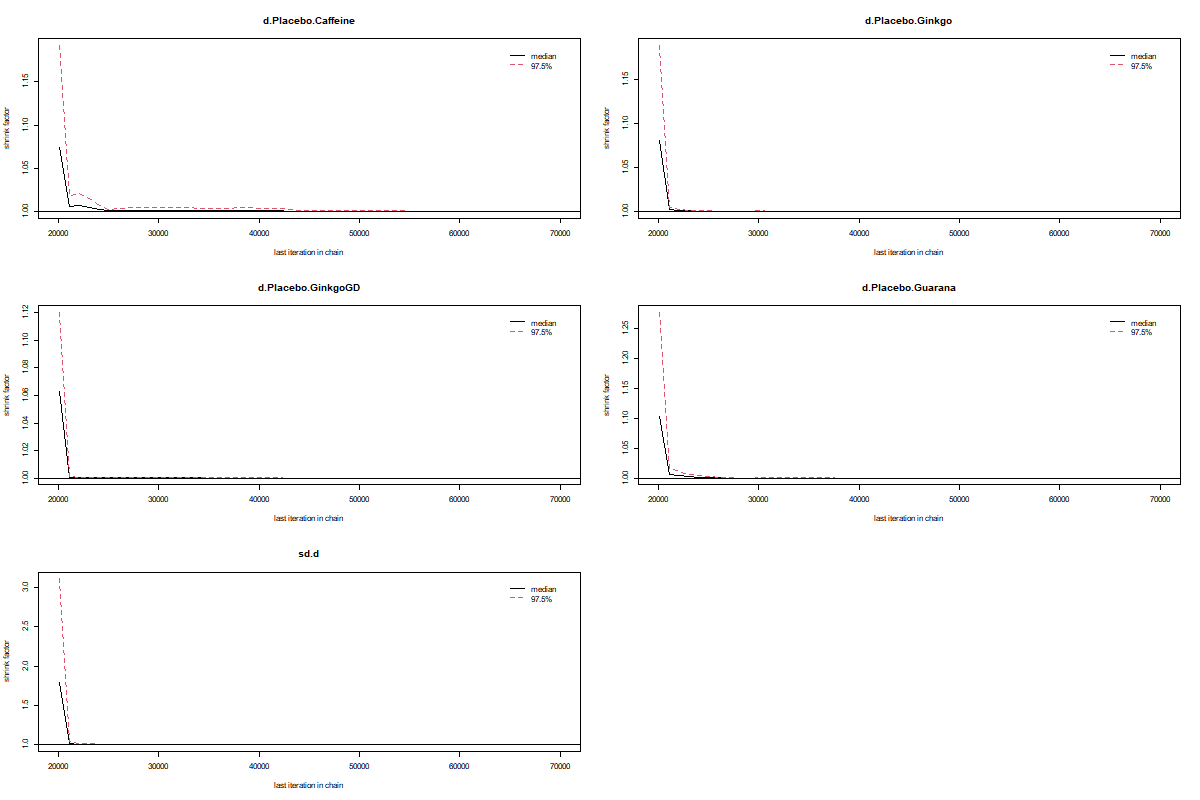


**Figure5B Gelman-Rubin convergence diagnostics for Language.**
