# Supplementary material for: The effect of plant active substances on cognitive function in healthy older adults: a systematic review and network meta-analysis of randomized controlled trials
Source: Front Pharmacol. 2026 Jan 20;16:1672171. doi: 10.3389/fphar.2025.1672171 (PMC12864429; doi:10.3389/fphar.2025.1672171)
Supplement: Supplementary file 3 [file Table1.docx]

**Supplementary Table1.** Search strategy on PubMed

| Search | PUBMED |
| --- | --- |
| #1 | ((((((Cognition[MeSH Terms]) ) OR (Cognitions[Title/Abstract])) OR (Cognitive Function[Title/Abstract])) OR (Cognitive Functions[Title/Abstract])) OR (Function, Cognitive[Title/Abstract])) OR (Functions, Cognitive[Title/Abstract]) |
| #2 | (((((((((((((Ginkgo biloba[MeSH Terms])) OR (Ginkgo[Title/Abstract])) OR (Ginkgos[Title/Abstract])) OR (Ginko[Title/Abstract])) OR (Maidenhair Tree[Title/Abstract])) OR (Maidenhair Trees[Title/Abstract])) OR (Tree, Maidenhair[Title/Abstract])) OR (Trees, Maidenhair[Title/Abstract])) OR (Gingko biloba[Title/Abstract])) OR (Gingko[Title/Abstract])) OR (Gingkos[Title/Abstract])) OR (Ginkgophyta[Title/Abstract])) OR (Ginkgo biloba extract[Title/Abstract]) |
| #3 | ((Ashwagandha[MeSH Terms]) OR (Withania[MeSH Terms])) OR (Withania somnifera[Title/Abstract]) |
| #4 | ((Bacopa monnieri[Title/Abstract]) OR (Bacopa monniera[Title/Abstract])) OR (Bacopa[Title/Abstract]) |
| #5 | (((((((((((((((((((Panax[MeSH Terms]) ) OR (Ninjin[Title/Abstract])) OR (Schinseng[Title/Abstract])) OR (Ginseng[Title/Abstract])) OR (Ginsengs[Title/Abstract])) OR (Jen Shen[Title/Abstract])) OR (Jen Shens[Title/Abstract])) OR (Shen, Jen[Title/Abstract])) OR (Renshen[Title/Abstract])) OR (Shinseng[Title/Abstract])) OR (Panax ginseng[Title/Abstract])) OR (Korean Red Ginseng[Title/Abstract])) OR (Ginseng, Korean Red[Title/Abstract])) OR (Korean Red Ginsengs[Title/Abstract])) OR (Red Ginseng, Korean[Title/Abstract])) OR (Korean Ginseng[Title/Abstract])) OR (Korean Ginsengs[Title/Abstract])) OR (Ginseng, Korean[Title/Abstract])) OR (American ginseng[Title/Abstract]) |
| #6 | (((((((Caffeine[MeSH Terms]) OR (1,3,7-Trimethylxanthine[Title/Abstract])) OR (No Doz[Title/Abstract])) OR (Caffedrine[Title/Abstract])) OR (Coffeinum N[Title/Abstract])) OR (Dexitac[Title/Abstract])) OR (Vivarin[Title/Abstract])) OR (Quick-Pep[Title/Abstract]) |
| #7 | (((((Curcumin[MeSH Terms]) OR (Curcumin Phytosome[Title/Abstract])) OR (Phytosome, Curcumin[Title/Abstract])) OR (Diferuloylmethane[Title/Abstract])) OR (Turmeric Yellow[Title/Abstract])) OR (Yellow, Turmeric[Title/Abstract]) |
| #8 | (((Salvia[MeSH Terms]) OR (Salvias[Title/Abstract])) OR (Salvia officinalis-[Title/Abstract])) OR (salvia officinalis leaf extract-[Title/Abstract]) |
| #9 | (((((((Rosmarinus[MeSH Terms]) OR (Rosemary Plant[Title/Abstract])) OR (Plant, Rosemary[Title/Abstract])) OR (Plants, Rosemary[Title/Abstract])) OR (Rosemary Plants[Title/Abstract])) OR (Rosmarinus officinalis[Title/Abstract])) OR (officinalis, Rosmarinus[Title/Abstract])) OR (Rosmarinus officinali[Title/Abstract]) |
| #10 | (((((((((((Blueberry Plants[MeSH Terms]) OR (Plant, Blueberry[Title/Abstract])) OR (Plants, Blueberry[Title/Abstract])) OR (Blueberry Plant[Title/Abstract])) OR (Blueberry[Title/Abstract])) OR (Blueberries[Title/Abstract])) OR (Vaccinium corymbosum[Title/Abstract])) OR (Vaccinium angustifolium[Title/Abstract])) OR (Vaccinium virgatum[Title/Abstract])) OR (Vaccinium ashei[Title/Abstract])) OR (Vaccinium uliginosum[Title/Abstract])) OR (blueberry extract[Title/Abstract]) |
| #11 | ((((((((Avena[MeSH Terms]) OR (Oats[Title/Abstract])) OR (Oat[Title/Abstract])) OR (Avena sativa[Title/Abstract])) OR (Cultivated Oat[Title/Abstract])) OR (Cultivated Oats[Title/Abstract])) OR (Oat, Cultivated[Title/Abstract])) OR (Oats, Cultivated[Title/Abstract])) OR (Green oat[Title/Abstract]) |
| #12 | (((((((((Melissa[MeSH Terms]) OR (Melissas[Title/Abstract])) OR (Melissa officinalis[Title/Abstract])) OR (Melissa officinali[Title/Abstract])) OR (officinalis, Melissa[Title/Abstract])) OR (Lemon Balm[Title/Abstract])) OR (Balm, Lemon[Title/Abstract])) OR (Balms, Lemon[Title/Abstract])) OR (Lemon Balms[Title/Abstract])) OR (Melissa officinalis leaf extract[Title/Abstract]) |
| #13 | ((((((((Tea[MeSH Terms]) OR (Green Tea[Title/Abstract])) OR (Green Teas[Title/Abstract])) OR (Tea, Green[Title/Abstract])) OR (Teas, Green[Title/Abstract])) OR (Black Tea[Title/Abstract])) OR (Black Teas[Title/Abstract])) OR (Tea, Black[Title/Abstract])) OR (Teas, Black[Title/Abstract]) |
| #14 | (((((((((((((((((((((((((Prunus avium[MeSH Terms]) OR (Sweet Cherry Tree[Title/Abstract])) OR (Cherry Trees, Sweet[Title/Abstract])) OR (Cherry Tree, Sweet[Title/Abstract])) OR (Sweet Cherry Trees[Title/Abstract])) OR (Trees, Sweet Cherry[Title/Abstract])) OR (Tree, Sweet Cherry[Title/Abstract])) OR (Prunus serotina[Title/Abstract])) OR (Black Cherry[Title/Abstract])) OR (Black Cherries[Title/Abstract])) OR (Cherries, Black[Title/Abstract])) OR (Cherry, Black[Title/Abstract])) OR (Prunus cerasus[Title/Abstract])) OR (Pie Cherry[Title/Abstract])) OR (Cherries, Pie[Title/Abstract])) OR (Cherry, Pie[Title/Abstract])) OR (Pie Cherries[Title/Abstract])) OR (Sour Cherry[Title/Abstract])) OR (Cherries, Sour[Title/Abstract])) OR (Cherry, Sour[Title/Abstract])) OR (Sour Cherries[Title/Abstract])) OR (Sweet Cherries[Title/Abstract])) OR (Cherries, Sweet[Title/Abstract])) OR (Cherry, Sweet[Title/Abstract])) OR (Sweet Cherry[Title/Abstract])) OR (cherry extract[Title/Abstract]) |
| #15 | (((Hericium[MeSH Terms]) OR (hericium erinaceus[Title/Abstract])) OR (Hericium erinaceus Extract[Title/Abstract])) OR (Lion's Mane Mushroom[Title/Abstract]) |
| #16 | ((((((Vitis[MeSH Terms]) OR (Grapes[Title/Abstract])) OR (Grape[Title/Abstract])) OR (Raisins[Title/Abstract])) OR (Raisin[Title/Abstract])) OR (Vitis vinifera[Title/Abstract])) OR (Grape Seed Extract[Title/Abstract]) |
| #17 | ((((Rhodiola[MeSH Terms]) OR (Rhodiola rosea[Title/Abstract])) OR (Roseroot[Title/Abstract])) OR (Roseroots[Title/Abstract])) OR (Rhodiola crenulata[Title/Abstract]) |
| #18 | ((((((((Paullinia[MeSH Terms]) OR (Paullinia cupana[Title/Abstract])) OR (cupana, Paullinia[Title/Abstract])) OR (Guarana[Title/Abstract])) OR (Paullinia pinnata[Title/Abstract])) OR (pinnata, Paullinia[Title/Abstract])) OR (Barbasco[Title/Abstract])) OR (Barbascos[Title/Abstract])) OR (guarana powder[Title/Abstract]) |
| #19 | (((((((((((((((((((Dioscorea[MeSH Terms]) OR (Yam[Title/Abstract])) OR (Yams[Title/Abstract])) OR (Dioscorea polystachya[Title/Abstract])) OR (Dioscorea batatas[Title/Abstract])) OR (Chinese Yam[Title/Abstract])) OR (Chinese Yams[Title/Abstract])) OR (Yam, Chinese[Title/Abstract])) OR (Yams, Chinese[Title/Abstract])) OR (Yams, Chinese[Title/Abstract])) OR (Shan Yao[Title/Abstract])) OR (Shan Yaos[Title/Abstract])) OR (Yao, Shan[Title/Abstract])) OR (Yaos, Shan[Title/Abstract])) OR (Dioscorea villosa[Title/Abstract])) OR (Wild Yam[Title/Abstract])) OR (Wild Yams[Title/Abstract])) OR (Yams, Wild[Title/Abstract])) OR (Yam, Wild[Title/Abstract])) OR (Dioscorea opposita[Title/Abstract]) |
| #20 | (((((((((((((((((valerian[MeSH Terms]) OR (Valerians[Title/Abstract])) OR (Valeriana[Title/Abstract])) OR (Valerianas[Title/Abstract])) OR (Valeriana officinalis[Title/Abstract])) OR (officinalis, Valeriana[Title/Abstract])) OR (Valeriana officinali[Title/Abstract])) OR (Heliotrope, Garden[Title/Abstract])) OR (Garden Heliotrope[Title/Abstract])) OR (Garden Heliotropes[Title/Abstract])) OR (Heliotropes, Garden[Title/Abstract])) OR (Valeriana extract[Title/Abstract])) OR (Valerianae extract[Title/Abstract])) OR (valerian extract[Title/Abstract])) OR (valerian root[Title/Abstract])) OR (valerianae radix[Title/Abstract])) OR (Valeriana officinalis root[Title/Abstract])) OR (Valeriana jatamansi root[Title/Abstract]) |
| #21 | ((((((((((((((((((#2) OR (#3)) OR (#4)) OR (#5)) OR (#6)) OR (#7)) OR (#8)) OR (#9)) OR (#10)) OR (#11)) OR (#12)) OR (#13)) OR (#14)) OR (#15)) OR (#16)) OR (#17)) OR (#18)) OR (#19)) OR (#20) |
| #22 | (#1) AND (#21) |
