# Supplementary material for: The effect of plant active substances on cognitive function in healthy older adults: a systematic review and network meta-analysis of randomized controlled trials
Source: Front Pharmacol. 2026 Jan 20;16:1672171. doi: 10.3389/fphar.2025.1672171 (PMC12864429; doi:10.3389/fphar.2025.1672171)
Supplement: Supplementary file 11 [file Supplementaryfile2.docx]

**Figure 6**  **Influence analysis for Learning and memory.**

**Figure 7**  **Influence analysis for Complex attention.**

**Figure 8**  **Influence analysis for Executive Function.**

**Figure 9**  **Influence analysis for Language.**

**Figure 10**  **Influence analysis for Perceptual-motor function**
